# Learning-induced gene expression in the heads of two *Nasonia* species that differ in long-term memory formation

**DOI:** 10.1186/s12864-015-1355-1

**Published:** 2015-03-10

**Authors:** Katja M Hoedjes, Hans M Smid, Elio GWM Schijlen, Louise EM Vet, Joke JFA van Vugt

**Affiliations:** Laboratory of Entomology, Plant Sciences Group, Wageningen University, P.O. box 8031, 6700AP Wageningen, The Netherlands; Department of Ecology and Evolution, University of Lausanne, Le Biophore, CH-1015 Lausanne, Switzerland; PRI Bioscience, Plant Research International, P.O. box 619, 6700AP Wageningen, The Netherlands; Department of Terrestrial Ecology, Netherlands Institute of Ecology (NIOO-KNAW), P.O. Box 50, 6700 AB Wageningen, The Netherlands; Department of Neurology, University Medical Center Utrecht, P.O. Box 85500, 3508 GA Utrecht, The Netherlands

**Keywords:** Antisense, Illumina Hi-Seq, Learning, Long-term memory, Strand-specific sequencing

## Abstract

**Background:**

Cellular processes underlying memory formation are evolutionary conserved, but natural variation in memory dynamics between animal species or populations is common. The genetic basis of this fascinating phenomenon is poorly understood. Closely related species of *Nasonia* parasitic wasps differ in long-term memory (LTM) formation: *N. vitripennis* will form transcription-dependent LTM after a single conditioning trial, whereas the closely-related species *N. giraulti* will not. Genes that were differentially expressed (DE) after conditioning in *N. vitripennis*, but not in *N. giraulti*, were identified as candidate genes that may regulate LTM formation.

**Results:**

RNA was collected from heads of both species before and immediately, 4 or 24 hours after conditioning, with 3 replicates per time point. It was sequenced strand-specifically, which allows distinguishing sense from antisense transcripts and improves the quality of expression analyses. We determined conditioning-induced DE compared to naïve controls for both species. These expression patterns were then analysed with GO enrichment analyses for each species and time point, which demonstrated an enrichment of signalling-related genes immediately after conditioning in *N. vitripennis* only. Analyses of known LTM genes and genes with an opposing expression pattern between the two species revealed additional candidate genes for the difference in LTM formation. These include genes from various signalling cascades, including several members of the Ras and PI3 kinase signalling pathways, and glutamate receptors. Interestingly, several other known LTM genes were exclusively differentially expressed in *N. giraulti*, which may indicate an LTM-inhibitory mechanism. Among the DE transcripts were also antisense transcripts. Furthermore, antisense transcripts aligning to a number of known memory genes were detected, which may have a role in regulating these genes.

**Conclusion:**

This study is the first to describe and compare expression patterns of both protein-coding and antisense transcripts, at different time points after conditioning, of two closely related animal species that differ in LTM formation. Several candidate genes that may regulate differences in LTM have been identified. This transcriptome analysis is a valuable resource for future in-depth studies to elucidate the role of candidate genes and antisense transcription in natural variation in LTM formation.

**Electronic supplementary material:**

The online version of this article (doi:10.1186/s12864-015-1355-1) contains supplementary material, which is available to authorized users.

## Background

The ability to learn and form memory and the underlying cellular processes are evolutionary conserved, but there is substantial natural variation in memory dynamics between species [1,2]. The opportunity to acquire new skills or adapt behaviour through learning is an important benefit and can increase fitness [3,4]. Memory formation can, however, be maladaptive when unreliable associations are formed [5]. In addition, the process of memory formation is energetically costly, depending also on the type of memory that is formed [6,7]. Therefore, variation in memory dynamics is considered to be an adaptation to specific ecological constraints [5].

Three main types of memory can be distinguished based on temporal expression and cellular pathways involved. Anaesthesia-sensitive memory (ASM), also known as short-term memory (STM), typically lasts from minutes up to an hour and is sensitive to disruptive treatments, such as a cold shock [8]. During the ASM phase, the formation of more stable and durable types of memory starts, a process called memory consolidation, and this process can take hours to days to complete [9]. Two main forms of consolidated memory are distinguished. Anaesthesia-resistant memory (ARM) typically lasts from hours to days and formation of this type of memory is thought to depend on changes in existing proteins [10]. Long-term memory (LTM) can last from days up to the entire lifetime of an animal. LTM formation is dependent on both transcription and translation and it is, therefore, considered the most costly type of memory [7]. As a result, many animal species require multiple conditioning trials, which are spaced in time, to induce LTM consolidation. Such repeated learning experiences allow animals to evaluate the information before investing in costly LTM [5]. A single conditioning trial or massed conditioning trials, i.e. multiple trials without or with a very short inter-trial interval, typically do not induce LTM formation, but result in the formation of ASM and ARM [8]. However, the number of trials required to form LTM differs, even between closely related species [2]. A number of insect species are known to consolidate LTM after a single conditioning trial [9,11]. Ecological factors, including the value of the appetitive or aversive stimulus and the reliability of the learned association, are considered decisive for the number of trials required to form LTM [12]. Very little is currently known about genetic and neural factors that are involved in natural variation in LTM formation.

We have studied the genetic basis of variation in LTM formation in the parasitic wasp *Nasonia vitripennis* and its closely related species *N. giraulti*. The genus *Nasonia* has emerged as a powerful model with unique opportunities for genetic studies on inter-species differences, because of the possibility to interbreed both species [13]. *Nasonia vitripennis* forms ASM, ARM and LTM after a single conditioning trial in which an odour is associated with the appetitive stimulus of a host to parasitize [14-16]. LTM is expressed 4 days after conditioning, as demonstrated by inhibition through transcription- and translation-inhibitors [16]. *Nasonia giraulti*, on the other hand, forms ASM and ARM after a single conditioning trial and this memory disappears within 2 days. Multiple spaced conditioning trials are required to induce long-lasting memory retention [16]. This difference in LTM formation between these two species, which is thought to be the result of differences in their ecology [14], provides excellent opportunities to study the genetic basis of LTM formation. A recent study, in which genes of *N. giraulti* where backcrossed into the genetic background of *N. vitripennis*, revealed two quantitative trait loci that underlie the difference in long-lasting memory retention between *N. vitripennis* and *N. giraulti* [17]. This study investigates differences in gene expression patterns related to LTM formation between the two *Nasonia* species, as a second approach to identify genes that are involved in the difference in LTM formation.

Conditioning will induce differential gene expression in *Nasonia* wasps, compared to the expression levels of unconditioned (i.e. naïve) wasps, as a result of learning, but also as a result of contact with the host and oviposition behaviour. Controls are necessary to distinguish learning from host- or odour induced gene expression. Comparing *N. vitripennis* that has been conditioned (host + odour) to *N. vitripennis* that has only had contact with the host is not a suitable comparison, as these wasps are known to learn multiple cues of the host environment upon host contact, including visual cues and information on the absence of odours [18,19]. Consequently, this comparison may not reveal differences in learning-induced gene expression. Exposure to the odour alone is also not a suitable control, as it could induce habituation, a non-associative form of learning, which may not occur when wasps experience both host and odour. For this reason, we determined conditioning-induced differential expression patterns, which reflects learning, but also contact to the host or odour, of the both *N. vitripennis* and *N. giraulti*. These wasps were subjected to an identical conditioning procedure, which provides a control for host or odour induced differential expression, as well as for gene expression related to ASM or ARM, which occurs in both species. By focussing only on conditioning-induced differentially expressed genes that are unique to *N. vitripennis* we identify the genes that are most likely to be involved in the difference in LTM between the two species.

The gene expression profiles of *N. vitripennis* and *N. giraulti* were analysed using Illumina HiSeq sequencing of RNA extracted from the heads of naïve and conditioned wasps. A strand-specific RNA-sequencing protocol was used to distinguish sense and antisense transcripts. Sequencing RNA strand-specifically is important considering that genes can be encoded on different strands of the DNA and a considerable part of these genes is known to overlap [20,21]. Strand-specific information, therefore, will improve the accuracy of the gene expression analysis. Also, antisense transcripts are known to have an important role in memory dynamics [22]. This is, to our knowledge, the first study of strand-specifically sequenced transcriptomes of insect brains.

RNA was isolated from naïve wasps, which were not conditioned, and from conditioned wasps at three time points after conditioning, i.e. immediately, 4 hours or 24 hours afterward (3 replicates per time point), in order to observe temporal patterns in gene expression during LTM formation. LTM formation is known to depend on at least two waves of transcriptional activity that occur during or shortly after conditioning, and several hours after conditioning respectively [23]. The three time points shortly after conditioning were chosen as this study aims to identify genes that are involved in the early and intermediate phases of LTM formation, which are expected to be decisive for LTM formation. Conditioning-induced gene expression was determined by comparing expression levels of conditioned wasps to the naïve wasps for both species separately, to control for naïve differences in gene expression between the two species. Differentially expressed genes after conditioning were subsequently analysed by (1) a GO enrichment analysis, to assess functional expression patterns, (2) analyses of known (long-term) memory genes, and (3) identification of genes with an opposing differential expression pattern in *N. vitripennis* and *N. giraulti*. Based on the combination of these analyses we describe temporal patterns of gene expression after conditioning for both species, as well as differences in conditioning-induced gene expression between the two species. Differentially expressed genes, especially genes with a known role in memory formation, that are unique to *N. vitripennis* or that have an opposed expression to *N. giraulti* (i.e. that are upregulated in one species and downregulated in the other species, or vice versa) were identified as promising candidate genes for regulating the difference in LTM formation between the two species. Considering that LTM formation is evolutionary conserved, the findings of the study may be applicable to other animal species as well.

## Results

### Transcriptome assembly and annotation

The results of the *de novo* transcriptome assembly (both filtered and unfiltered) are presented in Additional file 1: Table S1. The majority of the genes in the transcriptomes, respectively 74.7% for *N. vitripennis* and 73.0% for *N. giraulti*, had a single transcript. Genes with multiple splice variants (‘transcripts’) accounted, however, for 61.7% and 62.0% of all transcripts, respectively.

**Figure 1:**
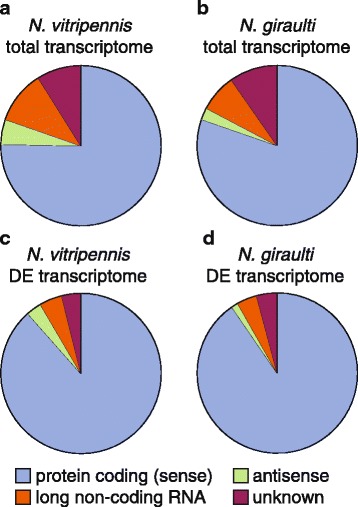
**The proportion of** **‘protein-coding** **(sense)** **’,** **‘antisense** **’, ‘** **long non**-**coding RNA** **’** **and** **‘** **unknown** **’** **is shown for** **(a)** ***N. vitripennis*** **total transcriptome** **(30223 transcripts),** **(b)** ***N. giraulti*** **total transcriptome** **(29641 transcripts),** **(c)** ***N. vitripennis*** **differentially expressed** **(DE)** **transcripts** **(2458 transcripts),** **and** **(d)** ***N. giraulti*** **DE transcripts** **(2220 transcripts)** **(DE compared to unconditioned expression).**

The percentages and average length of protein-coding (sense) transcripts, antisense transcripts, long non-coding RNA (lncRNA) and unknown transcripts are shown in Table 1 and Figure 1 (a-b). The head transcriptome of *N. giraulti* had a larger number of protein-coding transcripts than that of *N. vitripennis*, whereas it had half the amount of antisense transcripts. Also the fraction of lncRNA of *N. giraulti* was lower than that of *N. vitripennis*. A small portion of the lncRNA and unknown (i.e. misassembled or misassigned) transcripts contains a putative ORF, suggesting these might be (unknown) protein-coding genes.

**Table 1:** **Categories of transcripts in the transcriptomes**

**(a)** ***N. vitripennis***	**Total transcriptome**			**Total DE transcripts**			**DE transcripts per time point**		
	**Transcripts**		**Length**	**Transcripts**		**Length**	**0 h**	**4 h**	**24 h**
protein-coding (sense)	22760	75.3%	4076	2175	88.5%	3819	602	1109	1205
antisense	1525	5.0%		76	3.1%		29	41	37
*to a protein*	730	2.4%	2337	44	1.8%	3236	14	24	23
*to a sense transcript*	596	2.0%	1162	26	1.1%	802	13	16	10
*to both*	199	0.7%		6	0.2%		2	1	4
long non-coding RNA	3245	10.7%	1342	112	4.6%	1512	43	55	53
*with a putative ORF*	220	0.7%		10	0.4%		2	4	7
unknown	2693	8.9%	946	95	3.9%	1158	43	50	38
*with a putative ORF*	81	0.3%		4	0.2%		1	1	2
**Total**	**30223**		**3389**	**2458**		**3565**	**717**	**1255**	**1333**
(**b**) ***N. giraulti***									
protein-coding (sense)	23806	80.3%	4004	2008	90.5%	3336	340	854	1358
antisense	719	2.4%		30	1.4%		15	16	14
*to a protein*	154	0.5%	1165	4	0.2%	1254	4	1	0
*to a sense transcript*	529	1.8%	846	24	1.1%	561	11	14	12
*to both*	36	0.1%		2	0.1%		0	1	2
long non-coding RNA	2244	7.6%	1381	90	4.1%	1398	20	32	57
*with a putative ORF*	190	0.6%		12	0.5%		2	4	9
unknown	2872	9.7%	972	92	4.1%	1138	35	43	43
*with a putative ORF*	92	0.3%		2	0.1%		**2**	**0**	**0**
**Total**	**29641**		**3437**	**2220**		**3126**	**410**	**945**	**1472**

The number (and percentage of the total number of transcripts) and the average length (bp) of transcripts classified as ‘protein-coding (sense)’, ‘antisense’, ‘long non-coding RNA’ and ‘unknown’ are given for the total transcriptome, for all differentially expressed transcripts (compared to unconditioned expression) combined (transcripts that are differentially expressed at multiple time points are counted once), and for each of the three time points after conditioning of (a) *N. vitripennis* and (b) *N. giraulti*. For the ‘antisense’ transcripts, the number of transcripts with a hit to a protein, a sense transcript or both is also given. The number of transcripts with a putative ORF is given for ‘long non-coding RNA’ and ‘unknown’ transcripts.

### Conditioning-induced differential gene expression

**Figure 2:**
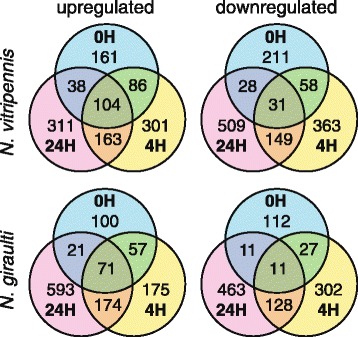
**The numbers of differentially expressed transcripts that are upregulated** **(left)** **or downregulated** **(right)** **at the indicated time points after conditioning** **(i.e. 0,** **4 and 24 h)** **are shown for** ***N. vitripennis*** **(top)** **and** ***N. giraulti*** **(bottom).**

The multi-dimension scaling plots of the biological coefficients of variation revealed that the gene expression data of the 3 biological replicates did not cluster per replicate; no clear clustering of samples per treatment was observed either (Additional file 1: Figure S1). Differential gene expression of the three time points after conditioning was determined in comparison to the expression levels of unconditioned (naïve) wasps for both species (Additional file 1: Figure S2). Information on these conditioning-induced differentially expressed (DE) transcripts is shown in Table 1 and Figure 1c-d. The proportion of sense transcripts was larger in the DE transcriptomes than in the complete transcriptomes, but still a number of antisense transcripts, lncRNA and unknown transcripts were differentially expressed. An analysis of DE transcripts of each time point after conditioning showed that the majority of the DE transcripts, i.e. 1759 transcripts of *N. vitripennis* (71.6%) and 1678 transcripts of *N. giraulti* (75.6%), were differentially expressed at only a single time point (Figure 2), which indicates substantial temporal differences in gene expression patterns after conditioning for both species.

**Figure 3:**
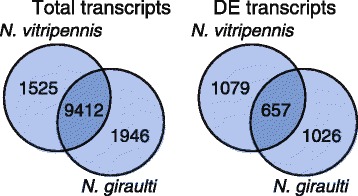
**The numbers of shared and unique protein-coding transcripts observed in the entire transcriptomes** **(left)** **and among differentially expressed** **(DE)** **transcripts** **(right)** **of** ***N. vitripennis*** **and** ***N. giraulti*** **are shown**.

The protein-coding transcripts of *N. vitripennis* and *N. giraulti* that had a hit to the *N. vitripennis* proteome were compared amongst each other to assess differences in gene expression between the two species. The majority of the transcripts of *N. vitripennis* and *N. giraulti*, 86.1% and 82.9% of the transcriptomes respectively, was observed in both species, which indicates a high level of similarity in transcripts expressed in the brains of both species. However, only 37.8% and 39.0% of the DE transcripts of *N. vitripennis* and *N. giraulti*, respectively, were differentially expressed in both species (Figure 3). This result suggests that there are substantial differences in conditioning-induced differential gene expression in *N. vitripennis* and *N. giraulti*. Results from analyses on DE transcripts are presented in the following paragraphs. Information on transcripts includes the *Drosophila* gene name (when available).

As mentioned, conditioning-induced differential expression was analysed in comparison to naïve expression levels for both *Nasonia* species separately, to control for innate differences in gene expression among the species. A comparison of naïve expression levels between the two species demonstrates that 1950 transcripts, of the 4275 transcripts that could be compared, differ in expression level (Additional file 1: Table S2). GO enrichment analyses of the innate differential expressed transcripts between both species reveal only a few enriched GO terms, related to several different processes, however (Additional file 1: Table S3), which suggests that this variation between both species does not focus on certain (learning related) processes or pathways.

### GO enrichment analysis of conditioning-induced differentially expressed genes

Differentially expressed protein-coding transcripts with a hit to the *N. vitripennis* proteome were analysed using GO enrichment analyses to provide insight into molecular functions of these genes. Additional file 1: Table S4 shows the complete lists of enriched GO terms for each of the analyses presented in this paragraph.

**Figure 4:**
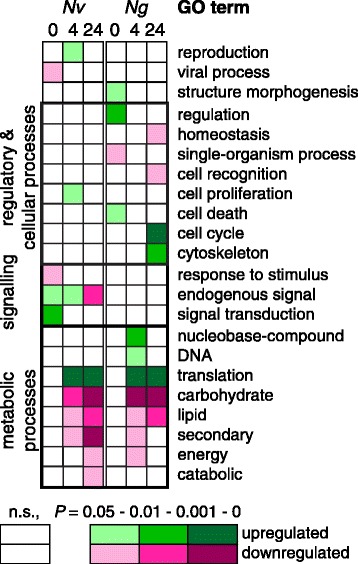
**The most specific significantly enriched GO terms in the category** **‘biological process’** **among upregulated** **(green)** **and downregulated** **(pink)** **transcripts 0 h,** **4 h,** **and 24 h after conditioning are presented for** ***N. vitripennis*** **(** ***Nv*** **,** **left)** **and** ***N. giraulti*** **(** ***Ng*** **,** **right).** The different shades of green and pink indicate how significantly the term is enriched.

Analyses of up- and downregulated transcripts that were differentially expressed immediately (0 hours), 4 hours or 24 hours after conditioning were done for both species separately. The most specific GO terms in the category ‘biological process’ are presented in Figure 4. There was no overlap in enriched GO terms between the two wasp species immediately after conditioning. Terms that indicate processes involved in signalling were observed exclusively in *N. vitripennis* both immediately after conditioning and at later time points, whereas a number of terms that indicate cell regulatory processes are unique for *N. giraulti*. Both *Nasonia* species had an overrepresentation of terms that indicate that translation of transcripts was upregulated at both 4 and 24 hours after conditioning. Terms that indicate metabolic processes, including lipid and carbohydrate metabolism were enriched in downregulated transcripts at both time points and in both species.

#### Signalling genes that are differentially expressed in N. vitripennis, but not in N. giraulti

Differences in GO enrichment between the two species were most pronounced immediately after conditioning and indicate processes involved in signal transduction or the response to stimuli in *N. vitripennis* only. We, therefore, analysed the genes underlying these enriched GO terms immediately after conditioning. A total of 71 transcripts (59 genes), were clustered in these GO terms (Additional file 1: Table S5). The DE transcripts include members of signalling cascades that are regulated by members of the Ras/Rho small G protein superfamily. Ras is known to activate the mitogen-activated protein kinase (MAPK) signalling pathway and the cAMP signalling cascade, which are both essential for LTM formation [24]. Rho signalling is known to be involved in dendritic remodelling through organization of the actin cytoskeleton and is also essential for long-term memory formation [25]. A total of 9 different transcripts involved in the Ras signalling cascade were upregulated or downregulated in *N. vitripennis*, but not in *N. giraulti*. Differentially expressed members of the Rho signalling cascade included a Rho GTPase-activating protein (*SLIT*-*ROBO*), and guanine nucleotide exchange factors (*still life* and *TRIO*) [26]. Ras-related protein Rab-32 [27] is upregulated in *N. vitripennis*, but downregulated in *N. giraulti*. Other genes with a known role in long-term memory formation included NMDA receptor 1 (upregulated) [28] and a metabotropic glutamate receptor (downregulated) [29].

### Analysis of known memory genes

**Figure 5:**
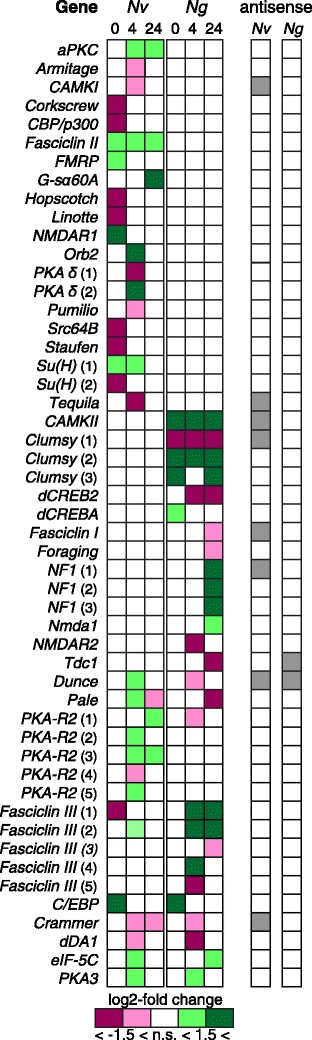
**Expression patterns of DE genes known to be involved in memory formation.** The log2-fold change shows if a transcript is upregulated (green, <1.5 or >1.5) or downregulated (pink, <−1.5 or > −1.5), n.s. = not significantly DE. Expression levels at 0 h, 4 h, and 24 h after conditioning are presented for *N. vitripennis* (*Nv*, left) and *N. giraulti* (*Ng*, right). For most genes, only a single transcript is DE and shown in this figure. Multiple DE transcripts of the same gene are indicated by (1), (2), etc. It is also indicated if an antisense transcript is present for each gene (grey = present, no color = not present).

A total of 78 genes with a known role in (long-term) memory formation was studied and 37 of these genes were observed to be differentially expressed after conditioning in *N. vitripennis* (18), *N. giraulti* (10) or both (9) (Additional file 1: Table S6 and Figure 5). Differential expression was observed in various signaling cascades that are involved in LTM formation in both *Nasonia* species.

The cAMP-signalling cascade is an important cascade in the formation of both ASM and LTM [8]. A cAMP phosphodiesterase (*dunce*) was upregulated in *N. vitripennis*, but downregulated in *N. giraulti*. Transcription factors cAMP response element binding protein A and B (i.e. *dCREBA* and *dCREB2*), which are critically involved in LTM formation [30,31], were not differentially expressed in *N. vitripennis*, but were respectively up- and downregulated in *N. giraulti*. A transcription co-activator of the *Notch* pathway (*Su*(*H*), the *Drosophila* homolog of *RBP*-*j*) which is critical for LTM formation [32], was upregulated only in *N. vitripennis*. Among the downregulated genes in *N. vitripennis* were *pumilio* and *staufen*, both involved in the subcellular localization of mRNA translation [33].

### Analysis of genes with an opposing differential expression pattern

A total of 87 genes were differentially expressed in both *Nasonia* species, but in opposite direction (Additional file 1: Table S7). Several of these genes have a function in metabolic processes, which is reflected by a GO enrichment analysis (Additional file 1: Table S7), but also a number of known memory genes were observed. These include a Rac GTPase-activating protein (downregulated in *N. vitripennis*) [34], the cAMP phosphodiesterase *dunce*, and phosphatidylinositol 3-kinase (PI3KC3) [35] (both upregulated in *N. vitripennis*). In addition, two other genes that are known to interact with PI3-kinase were identified (Glycosylphosphatidylinositol anchor attachment 1 protein and 1-phosphatidylinositol-4.5-bisphosphate phosphodiesterase; both downregulated in *N. vitripennis*).

### Analysis of antisense transcripts

Antisense transcripts with a hit to the *N. vitripennis* proteome were analysed using GO enrichment analyses to provide insight into the molecular functions of their sense transcripts (Additional file 1: Table S8). The two categories of antisense transcripts were analysed separately: ‘antisense2protein’ transcripts that have a hit to a *N. vitripennis* protein and ‘antisense2sense’ transcripts that have a hit to a sense transcript only (and this sense transcript must have a hit to a *N. vitripennis* protein). The number of DE antisense transcripts was too small to perform a GO enrichment analysis.

#### GO enrichment analyses of antisense transcripts

A diverse group of overrepresented GO terms was observed in antisense transcripts of both wasp species, which suggests that antisense transcripts play a role in various processes (Additional file 1: Table S9). For *N. vitripennis* these terms concerned processes involved in lipid and DNA metabolism and cytoskeleton organisation in antisense2protein transcripts and behaviour in antisense2sense transcripts. For *N. giraulti*, terms concerning processes involved in gene expression were observed in antisense2protein transcripts and cell signalling, response to an abiotic stimulus, organelle organization, growth, anatomical structure morphogenesis and symbiosis in antisense2sense transcripts. The terms behaviour, cell signalling and response to an abiotic stimulus may implicate that part of these antisense transcripts were involved in synaptic processes or memory formation.

#### Differentially expressed antisense transcripts

A small number of DE antisense transcripts was observed: 76 in *N. vitripennis* and 29 in *N. giraulti* (Additional file 1: Table S9). Only two proteins had DE antisense transcripts in both species, but the function of these proteins is unknown. Although the other proteins with DE antisense transcripts were not similar in both species, they were involved in similar functions, i.e. gene expression and signalling. Gene expression had an emphasis on chromatin remodelling enzymes, like DNA polymerase *η* and chromatin assembly factor *Caf1* in *N. vitripennis*. Observed signalling proteins included a Ras-related small GTPase (*Ras*-*26*), which had a DE antisense transcript in *N. vitripennis* [36]. One DE antisense transcripts aligned to a subunit of phosphatidylinositol 3-kinase, which is known to be involved in memory formation [35].

#### Antisense transcripts that align to known memory genes

Analysis of the 78 known memory genes (Additional file 1: Table S6), described in the previous paragraph, revealed that 14 of these genes had antisense transcripts (Additional file 1: Table S8). Antisense transcripts that align to a known memory gene (that was differentially expressed after conditioning) are presented in Figure 5. In addition, the kinase *S6KII*, glutamate receptor *GluCl*, 14-3-3 zeta protein *leonardo*, an octopamine receptor (*Octß2R*) and Rap GTPase activating protein *radish* had an antisense transcript. Only the antisense transcript of *fasciclin* 1 was differentially expressed after conditioning. For the majority of these genes, an antisense transcript was detected in only one species (9 from *N. vitripennis* only, 3 from *N. giraulti* only). For only two of the memory genes (*dunce* and *S6KII*), an antisense transcript in both *N. vitripennis* and *N. giraulti* was observed. All 14 of the antisense transcripts aligned to a sense transcript, whereas two antisense transcripts aligned to a protein as well (i.e. the other 12 transcripts aligned to a non-protein-coding section of the sense transcript, potentially the UTRs). This result corresponded to results from the GO enrichment analyses, which indicated that memory-related terms concerning behaviour, cell signalling and response to an abiotic stimulus were overrepresented in antisense2sense transcripts only. It suggests that antisense transcripts related to memory formation align more often to the untranslated region rather than to the protein-coding region of a gene.

## Discussion

This study describes the gene expression patterns in the heads of *N. vitripennis* and *N. giraulti*, two closely related parasitic wasp species that differ in LTM formation after a single conditioning trial.

### Differential gene expression after conditioning

Of all transcripts in the heads of *N. vitripennis* and *N. giraulti*, respectively 8.1% and 7.5% were differentially expressed at one or multiple time points after conditioning, compared to the naïve expression levels. .

#### Temporal patterns of gene expression after conditioning

Our results demonstrated surprisingly little overlap in differentially expressed genes between the two species, but GO enrichment analyses did demonstrate several similarities in the molecular functions of these DE genes. These analyses also demonstrate distinct temporal patterns in the molecular functions of differentially expressed transcripts at the three time points after conditioning. For both wasp species, the enriched GO terms observed immediately after conditioning (mostly signalling processes in *N. vitripennis* and cell regulatory processes in *N. giraulti*), had very little overlap with those at 4 or 24 hours after conditioning. Also, the most pronounced differences between the two species were observed immediately after conditioning, a procedure that lasts in total 1.5 hour in our experiment. This result suggests that this early differential gene expression may be decisive for whether LTM formation is initiated after a single conditioning trial or not, as also indicated by previous studies [23]. However, several differences between the species are also observed at later time points, which were expected as LTM formation takes 4 days to complete in *Nasonia*. Of the known memory genes, 13 transcripts were differentially expressed immediately after conditioning in *N. vitripennis*, 21 after 4 hours and 7 after 24 hours. For *N. giraulti* these numbers are respectively 6, 14, and 17. The enriched GO terms at 4 and 24 hours after conditioning were largely overlapping and indicate processes involved in metabolism and translation in both species.

#### Differentially expressed genes that are unique for N. vitripennis: candidate genes for LTM formation

Among the considerable differences in expression patterns between the two *Nasonia* species after conditioning are well-known signalling and memory genes from various genetic pathways. Both up- and downregulation of genes with a known role in (long-term) memory formation was observed in *N. vitripennis*, which may point to activation of positive regulatory mechanisms, as well as deactivation of LTM inhibitory mechanisms [37]. Interesting observations include the pronounced differential expression of genes that are part of the Ras and Rho signalling cascades, which was observed immediately after conditioning in *N. vitripennis*, but much less in *N. giraulti*. A couple of glutamate receptors were differentially expression in *N. vitripennis* only, whereas others were differentially expressed in *N. giraulti* only. Finally, an opposite expression pattern was observed for three genes involved in PI3 kinase signalling between *N. vitripennis* and *N. giraulti*. Although our study is the first to compare gene expression patterns after conditioning between animals that differ in memory performance, two other studies have made such a comparison between naïve animals. Pravosudov *et al*. [38] report on two populations of chickadees that differ in spatial memory performance and Armbrecht *et al*. [39] compared control mice and mice with impaired memory performance. These studies reported differences in gene expression in various genes, including genes in the Ras signalling pathway and glutamate receptors in the chickadees, and genes in the Ras and PI3K signalling pathways in mice. This may indicate that these genes have an evolutionary conserved role in regulating natural variation in (long-term) memory performance and makes these genes promising candidates for further studies.

Some of the differentially expressed genes that were observed only in *N. vitripennis* may not be involved in LTM formation, but rather in ARM formation. In *N. vitripennis* two types of ARM are distinguished [15]. A short lasting type is observed from an hour up to at least a day after conditioning and this type is likely also formed in *N. giraulti*. A second type of long lasting ARM, which can be blocked by ethacrynic acid, is observed at 72 hours after conditioning in *N. vitripennis*, but not in *N. giraulti* [19]. The kinase *aPKC* is known to be involved in ARM formation [8], and was upregulated in *N. vitripennis*, but not in *N. giraulti*. The observed differential expression of *aPKC* may be related to the formation of the long-lasting type of ARM in *N. vitripennis*.

#### Differentially expressed genes that are unique for N. giraulti: LTM inhibitory mechanisms

A number of genes with a well-described role in LTM formation was differentially expressed in *N. giraulti* only, for example two *CREB* transcription factors and GTP cyclohydrolase 1, an enzyme involved in dopamine neurotransmitter synthesis [31,40,41], even though this species does not form LTM after a single conditioning trial. These differentially expressed genes may be part of an active inhibitory mechanism of LTM formation in this species. Focussing on potential LTM inhibiting genes to explain natural variation in memory formation among species may, therefore, be another interesting approach for further studies.

#### Non-LTM induced gene expression

Our results have shown substantial differences in differential gene expression after conditioning between *N. vitripennis* and *N. giraulti*, even though the two species were subjected to an identical conditioning protocol and both formed ASM and ARM. A recent proteome analysis of *Drosophila* brains after odour-electric shock conditioning indicates substantial changes in protein expression, regardless of whether ARM or LTM was formed, whereas relatively few differences in protein expression were observed in flies that formed LTM, compared to ones that formed ARM [42]. Compared to this study, our substantial differences between the two species are surprising and it is possible that some of the observed differences in conditioning-induced differential gene expression rather reflect species-specific differences in the response to the host than the difference in LTM formation.

Although we have described several differences, there are also hundreds of differentially expressed genes that are observed in both species. In addition, GO enrichment analyses indicate pronounced changes in translation and metabolism after conditioning, especially after 4 and 24 hours, in both species. This overlap may be the result of gene expression involved in ASM or ARM formation, memory types that do not depend on transcription during or shortly after conditioning [8], but which may induce differential gene expression afterwards. The proteome analysis of *Drosophila* brains, mentioned above, indicates substantial changes in protein expression related to metabolism, regardless of whether ARM or LTM was formed, which seems to suggest a role for metabolism-related pathways in memory formation in general [42].

Part of the overlapping conditioning-induced differential gene expression may also be related to contact with the host, which was similar in both *Nasonia* species. During conditioning, the wasps will touch, evaluate and typically also feed from the host haemolymph, which induces the formation of eggs that are required for future oviposition. A recent study in *N. vitripennis* females indicated downregulation of various metabolic processes in ovipositing females compared to resting females [43]. The observed changes in metabolic pathways, which were observed in both *N. vitripennis* and *N. giraulti* in this study, may therefore also be related to oviposition behaviour.

### Alternative splicing

Alternative splicing was detected in large numbers of multi-exon genes and is known to be important for protein function, especially in neuronal genes [44,45]. For the transcription factor *CREB*, important for LTM formation, both inhibiting and activating transcript variants have been described and the balance of different transcript variants determines the number of trials required to initiate LTM consolidation in *D. melanogaster* [31]. Different splice variants of fragile X mental retardation protein (*FMRP*) in *D. melanogaster* are thought to be involved in ASM and LTM, respectively [46]. Information on splice variants is, therefore, crucial for understanding gene functioning, but reliable and accurate determination of splice variants is challenging due to the small length of HiSeq reads that were analysed in this study. Multiple splice variants were detected for approximately 25% of all genes in the (head) transcriptomes of *N. vitripennis* and *N. giraulti* and for the majority of the studied memory genes (62 out of 78). However, for most DE genes with multiple splice variants, only a single splice variant was differentially expressed. Examples of DE genes of which more than one splice variant was differentially expressed are the transcription coactivator *Su*(*H*), protein kinase A-R2, protein kinase Cδ, glutamate receptor *clumsy*, and the neural cell adhesion molecule *fasciclin 3*. Different splice variants of the same gene can be up- or downregulated. Studies on the role of individual splice variants of candidate genes are necessary to unravel if and how these genes are involved in the regulation of LTM formation in *Nasonia* wasps.

### Non-coding sequences

RNA sequences that do not encode proteins are thought to have important roles in the regulation of gene expression [47,48]. The strand-specific sequencing protocol enabled distinguishing sense- and antisense transcripts, which ensures a more accurate determination of gene expression. It also provided, for the first time, the opportunity to study conditioning-induced expression patterns of antisense transcripts. We focused on antisense RNA, although other lncRNAs (>200 bp in length), that aligned to genomic DNA, were also distinguished. These lncRNAs were not described in detail as their function is difficult to interpret from a gene expression analysis without further functional studies. We did not study small non-coding RNAs, because they were not sequenced with our methodology.

Antisense transcripts are thought to regulate transcription or translation of the protein-coding gene originating from the opposite DNA strand, but also of neighbouring genes; they can regulate transcription initiation, transcription elongation, alternative splicing, and affect mRNA stability and translation efficiency [49]. Antisense transcripts can affect chromatin structure and DNA methylation, which are also known to be important for alternative splicing and transcription regulation in the brain and for memory formation specifically [49]. A total of 5.0% (1525) and 2.4% (719) of all transcripts were classified as antisense transcripts in *N. vitripennis* and *N. giraulti*, respectively. The actual number of antisense transcripts may be higher, because only transcripts with a high percentage of alignment length and identity were classified as antisense transcripts and others were classified as lncRNA or unknown transcripts. An interesting observation is that 47.9% (730) and 21.4% (154) of these *N. vitripennis* and *N. giraulti* antisense transcripts, respectively, only aligned to a known protein, but not to a sense transcript, which suggests that these genes had been silenced. GO enrichment analyses of antisense transcripts revealed an overrepresentation of genes involved in behaviour and signalling, which hints towards a role in the regulation of memory formation related genes and antisense transcripts were observed for 14 out of 78 known memory genes that were studied. In addition, differential expression of antisense transcripts after conditioning had been observed. Although the significance of these observations remains to be investigated, they hint towards a role of antisense transcripts in the regulation of long-term memory formation.

## Conclusion

Our transcriptome analysis is the first to provide an extensive overview of conditioning-induced differential expression patterns of both protein-coding and antisense transcripts, in the heads of two *Nasonia* parasitic wasp species, which differ in the formation of LTM. Although we identified the most promising candidate genes for this difference in LTM by focussing on genes with a different conditioning-induced expression pattern between the two *Nasonia* species, further functional studies are required to confirm that these genes indeed have a role in variation in LTM formation. RNAi can be used to manipulate gene expression levels and investigate if and how candidate genes are involved in memory formation [50]. Our study is a valuable resource for such future studies on the genetic basis of variation in (long-term) memory. Considering that learning and memory formation are evolutionary conserved, our results may be applicable to other species and may provide novel insights for studies on neurodegenerative diseases in humans, in which known memory genes are involved as well.

## Methods

### Insects

*Nasonia vitripennis* (strain AsymCx) and *N. giraulti* (strain RV2x(U)) were used in the experiments. These strains are completely homozygous and have a sequenced genome [13]. Wasps were reared on *Calliphora vomitoria* pupae as described by Hoedjes *et al*. [14]. Female wasps were collected on the day of emergence, were provided honey and water in a polystyrene rearing vial, and were kept in a climate cabinet at 25°C and a photoperiod of 16:8 (L/D). This study involved non-regulated (invertebrate) species and, therefore, was exempt from ethical approval.

### Conditioning procedure

Female wasps were conditioned using a Pavlovian conditioning assay in which an odour (chocolate) is associated with the appetitive stimulus of a host (*C. vomitoria* pupa) as described by Hoedjes *et al*. [14]. Briefly, wasps were individually given two host pupae (appetitive unconditioned stimulus, US) in a well of a 12 well-microtiter plate in the presence of chocolate odour (the conditioned stimulus, CS+). Wasps were allowed to drill into the pupae and perform host feeding for 1 hour. Oviposition does not take place during this period. Wasps that did not initiate drilling within 30 minutes (~5-10%) were removed from the experiment. After the 1-hour period, the wasps were gently removed from the hosts and transferred to a clean rearing vial. After a 15-minute resting period, wasps were exposed to vanilla odour (CS) for another 15 minutes without an appetitive or aversive stimulus present, which enhances memory performance. After this conditioning trial, the wasps were transferred to a rearing vial with access to honey and water, and were kept in a climate cabinet as described above. Naïve wasps, from the same age and batch as the conditioned wasps, were collected and transferred to rearing vials directly, without exposing them to either hosts or odour. These wasps served as a control to which the gene expression levels of the conditioned wasps were compared (i.e. to determine conditioning-induced differential gene expression). Both *N. vitripennis* and *N. giraulti* were conditioned using this protocol. Three groups of 30 wasps were (individually) conditioned per species at the same time. This was repeated 3 times on different days.

### Sample preparation and RNAseq

Groups of 30 wasps were collected for RNA isolation (1) immediately after conditioning, (2) 4 hours after conditioning or (3) 24 hours after conditioning and (4) without conditioning (naïve controls). The naïve controls were collected for RNA isolation immediately after the other wasps had been conditioned. We did not collect unconditioned wasps at the other time points after conditioning, as age-related differences in gene expression in the 24 hours after conditioning were expected to be small, compared to conditioning-induced differential gene expression. Furthermore, the subsequent analyses, which compared the two *Nasonia* species, provide a control for potential differences in age-related gene expression among time points.

Wasps were frozen in liquid nitrogen; heads were cut off with a scalpel and collected in a 1.5 ml microcentrifuge tube, which was stored in liquid nitrogen. RNA was extracted from the heads using the RNeasy Micro Kit (Qiagen, Antwerp, Belgium) according to instructions of the manufacturer. A total of 3 biological replicates were collected for each of the three treatments and unconditioned controls, resulting in 12 samples per *Nasonia* species. RNA quantity and integrity was measured using a 2100 Bioanalyzer (Agilent Technologies, Amstelveen, The Netherlands). The RNA concentration ranged from 270 – 650 ng/μl and the RNA integrity number (RIN) was between 9.7 and 10 [51].

One microgram total RNA was used for mRNA isolation and subsequent RNAseq library preparation following the TruSeq Stranded mRNA Sample Preparation Protocol (Illumina). In short, mRNA was isolated using oligo dT beads and chemically fragmented prior to first strand cDNA synthesis using random hexamer primers. Strand specificity was achieved by replacing dTTP with dUTP during Second Strand synthesis and the addition of transcription inhibitor Actinomycin D to the First Strand Master Mix. Obtained cDNA fragments were used for 3’adenylation and adapter ligation using 24 different barcoded adapters, one for each library. Adapter-ligated cDNA was amplified using 15 PCR cycles. Quality control of libraries was done using Agilent Bioanalyzer2100 DNA 1000 assays. Quantification was performed using Quant-iT PicoGreen dsDNA reagent (Molecular Probes, Invitrogen) and a fluorescence plate reader system (Tecan XFluor). Equimolar amounts of all 24 libraries were pooled together and were applied on two lanes together with Illumina V3 reagents. Paired-end 100 bp sequencing was performed on a HiSeq2000 instrument. De-multiplexing of obtained sequences was done using CASAVA 1.8.1. software.

### Transcriptome assembly

All reads were quality filtered and adapter trimmed using cutadapt (version 0.9.5), options: −O 10, −n 3, −q 10. Data were then filtered using fastq-mcf, options: −k 5, −q 20, −l 50.

The reads of all *N. vitripennis* samples were pooled to assemble the transcriptome *de novo* using Trinity (version r2013-02-15, option: −-SS_lib_type RF) [52]. The same was done for *N. giraulti*. The assembled transcripts have names that consist of three parts, for example comp100_c0_seq1, of which the first two parts define the “gene” name. All transcripts from one “gene” were considered to be alternative splice variants of that gene, for example comp100_c0_seq1 and comp100_c0_seq2. Transcripts smaller than 200 bp and those that had little read support were removed from the transcriptome. The latter was done by first mapping the unfiltered reads of each sample individually back to the transcriptome using bowtie (version 0.12.7, options: −n 2, −e 99999999, −l 25, −3 0, −a, −m 200, −I 1, −X 1000, −-nofw) and quantifying the mapped reads using eXpress (version 1.3.1). Then, the rounded effective read counts per transcript were analysed using R (version 2.15.2) and only transcripts with more than one read count per million (cpm) for at least 3 samples were kept.

### Annotation

Transcripts were annotated by aligning them to the *N. vitripennis* proteome (Nvit 2.0) or NCBI RefSeq nr database (sept-01-2013) using blastx (options: −max_target_seqs 1, −word_size 11, e-value 10), which is integrated in the Blast facility of the Centre for BioSystems Genomics (CBSG) and Wageningen University (created by Applied Bioinformatics, Plant Research International). Because the mRNA was sequenced strand specifically, the sense or antisense orientation of the aligned transcripts could be deduced.

The transcripts were first aligned to the *N. vitripennis* proteome. Transcripts that aligned to a protein with less than 60% protein alignment length were aligned to the NCBI RefSeq nr database. Protein-coding transcripts were defined as sense transcripts if they had more than 60% protein alignment length to a *N. vitripennis* protein or NCBI RefSeq nr database protein. The transcripts that did not align to a protein with more than 60% protein alignment length could be of different origin: (1) sense RNA encoding proteins not present in the published proteome databases, (2) sense RNA encoding proteins smaller than 60% of the complete protein, for example unknown small splice variants, (3) antisense RNA, (4) lncRNA, or (5) misassembled transcripts. Point (2) was addressed by also defining all transcript variants of a protein-coding (sense) transcript as sense transcripts, even if they were smaller than 60% if the complete protein. We defined antisense transcripts (3) as transcripts with an antisense orientation to a protein with more than 50% protein alignment length, or with an antisense orientation to a sense transcript with more than 80% antisense transcript alignment length and 95% sequence identity. Antisense transcripts that do not align to a protein, but only to a sense transcript likely have a hit to an untranslated region of that gene. Transcripts that were not categorized as sense or antisense transcripts but aligned to the *N. vitripennis* genome or NCBI RefSeq nt database with more than 80% alignment length and 95% sequence identity are suggested to be lncRNA (4). Transcripts without sense, antisense or long non-coding label were defined as ‘unknown’ (5) and may include misassembled transcripts, but also (anti)sense transcripts or lncRNA with insufficient alignment length or identity to known sequences. Putative open reading frames (ORFs) were determined for long non-coding and unknown transcripts using the script ‘transcripts_to_best_scoring_ORFs.pl’ from Trinity (options -m 30 -S). Putative ORFs were defined as an ORF with a 5’start and 3’end and minimally 30 amino acids.

### Differential expression analysis

Conditioning-induced differentially expressed (DE) transcripts in the *N. vitripennis* and *N. giraulti* transcriptomes, compared to naïve expression levels, were identified using EdgeR (version 3.0.8). This analysis includes normalization for differences in length between genes. The rounded effective read counts of each sample, extracted from eXpress, were analysed using a GLM trended dispersion with Pearson correlation with eight degrees of freedom and *P* = 0.05, and also taking the replica effect into account. A Benjamini and Hochberg’s approach was applied for controlling the false discovery rate (FDR < 0.05). To assess inherent differences in gene expression between the two species, the differential gene expression of naïve *N. giraulti* compared to naïve *N. vitripennis* expression was identified as well (Additional file 1: Table S2 and Additional file 2).

Three complementary analyses were used to analyse the conditioning-induced differential gene expression patterns of *N. vitripennis* and *N. giraulti*. (1) Gene Ontology (GO) enrichment analyses were performed on the DE transcripts that aligned to the *N. vitripennis* proteome (Nvit 2.0) using the Blast2go GUI and a Fisher’s exact test, *P* < 0.05, in order to visualize expression patterns of functional clusters of genes. GO terms were linked to the Nvit 2.0 proteome using Blast2GO as described on [53]. Generic GOSlim categories were used to limit the number of GO term categories (Gene Ontology Consortium, jan-10-2014). Enriched GO terms were compared between *N. vitripennis* and *N. giraulti* transcripts (Additional file 1: Table S4), for different time points after conditioning for each individual species, and for antisense transcripts. (2) The gene expression patterns of 78 genes that are known from literature to be involved in (long-term) memory formation were analysed for both species (Additional file 1: Table S4). The *Nasonia* homolog of a ‘memory’ gene was obtained by aligning the *Drosophila melanogaster* gene or protein sequence to the *N. vitripennis* genome or proteome using blastn or blastp. (3) Genes that were differentially expressed in *N. vitripennis* and *N. giraulti*, but in opposite direction, were identified and studied with a GO enrichment analysis (Additional file 1: Table S7).

### Availability of supporting information

The data sets supporting this Transcriptome Shotgun Assembly project are available in the DDBJ/EMBL/GenBank repository under the accessions GBEB00000000 (*N. vitripennis*) and GBEC00000000 (*N. giraulti*). The versions described in this paper are the first versions, GBEB01000000 (*N. vitripennis*) and GBEC01000000 (*N. giraulti*).

## Additional files

Additional file 1: Figure S1. Multi-dimension scaling plots of the biological coefficients of variation. **Figure S2.** Distribution of DE transcripts. **Table S1**. transcriptome analysis statistics. **Table S2.** Comparison of naïve gene expression levels in the heads of *N. vitripennis* and *N. giraulti*. **Table S3.** Enriched GO terms in genes that differ in expression levels between naïve *N. vitripennis* and *N. giraulti*. **Table S4.** Enriched GO terms in differentially expressed protein-coding transcripts. **Table S5.** DE transcripts involved in signalling in *N. vitripennis*. **Table S6.** Differential expression of known memory genes after conditioning. **Table S7.** Differentially expressed genes with an opposed expression pattern after conditioning in *N. vitripennis* and *N. giraulti*. **Table S8.** Enriched GO terms in antisense transcripts. **Table S9.** Antisense transcripts. Additional file 2:
**Comparison of naïve gene expression levels in the heads of** ***N. vitripennis*** **and** ***N. giraulti*** **(data belonging to Additional file** **1** **: Table S2).**

## References

[1] Brenowitz EA, Beecher MD (2005). Song learning in birds: diversity and plasticity, opportunities and challenges. Trends Neurosci.

[2] Hoedjes KM, Kruidhof HM, Huigens ME, Dicke M, Vet LEM, Smid HM (2011). Natural variation in learning rate and memory dynamics in parasitoid wasps: opportunities for converging ecology and neuroscience. Proc R Soc B.

[3] Papaj DR, Vet LEM (1990). Odor learning and foraging success in the parasitoid, *Leptopilina heterotoma*. J Chem Ecol.

[4] Raine NE, Chittka L (2008). The correlation of learning speed and natural foraging success in bumblebees. Proc R Soc B.

[5] Menzel R (1999). Memory dynamics in the honeybee. J Comp Physiol A.

[6] Laughlin SB (2001). Energy as a constraint on the coding and processing of sensory information. Curr Opin Neurobiol.

[7] Mery F, Kawecki TJ (2005). A cost of long-term memory in *Drosophila*. Science.

[8] Margulies C, Tully T, Dubnau J (2005). Deconstructing memory in *Drosophila*. Curr Biol.

[9] Smid HM, Wang GH, Bukovinszky T, Steidle JLM, Bleeker MAK, van Loon JJA (2007). Species-specific acquisition and consolidation of long-term memory in parasitic wasps. Proc R Soc B.

[10] Tully T, Preat T, Boynton SC, Del Vecchio M (1994). Genetic dissection of consolidated memory in *Drosophila*. Cell.

[11] Krashes MJ, Waddell S (2008). Rapid consolidation to a radish and protein synthesis-dependent long-term memory after single-session appetitive olfactory conditioning in *Drosophila*. J Neurosci.

[12] Kruidhof HM, Pashalidou FG, Fatouros NE, Figueroa IA, Vet LEM, Smid HM (2012). Reward value determines memory consolidation in parasitic wasps. PLoS One.

[13] Werren JH, Richards S, Desjardins CA, Niehuis O, Gadau J, Colbourne JK (2010). Functional and evolutionary insights from the genomes of three parasitoid *Nasonia* species. Science.

[14] Hoedjes KM, Steidle JLM, Werren JH, Vet LEM, Smid HM (2012). High-throughput olfactory conditioning and memory retention test show variation in *Nasonia* parasitic wasps. Genes Brain Behav.

[15] Schurmann D, Sommer C, Schinko APB, Greschista M, Smid H, Steidle JLM (2012). Demonstration of long-term memory in the parasitic wasp *Nasonia vitripennis*. Entomol Exp Appl.

[16] Hoedjes KM, Smid HM (2014). Natural variation in long-term memory formation among *Nasonia* parasitic wasp species. Behav Process.

[17] Hoedjes KM, Smid HM, Vet LEM, Werren JH: Introgression study reveals two quantitative trait loci involved in interspecific variation in memory retention among Nasonia wasp species. Heredity 2014, advance online publication: doi: 10.1038/hdy.2014.1066. 10.1038/hdy.2014.66 PMC4274617 25052416

[18] Oliai SE, King BH (2000). Associative learning in response to color in the parasitoid wasp *Nasonia* vitripennis (Hymenoptera: Pteromalidae). J Insect Behav.

[19] Schurmann D, Collatz J, Hagenbucher S, Ruther J, Steidle J (2009). Olfactory host finding, intermediate memory and its potential ecological adaptation in *Nasonia vitripennis*. Naturwissenschaften.

[20] Katayama S, Tomaru Y, Kasukawa T, Waki K, Nakanishi M, Nakamura M (2005). Antisense transcription in the mammalian transcriptome. Science.

[21] Sanna CR, Li WH, Zhang L (2008). Overlapping genes in the human and mouse genomes. BMC Genomics.

[22] Saab BJ, Mansuy IM (2014). Neuroepigenetics of memory formation and impairment: the role of microRNAs. Neuropharmacology.

[23] Barzilai A, Kennedy TE, Sweatt JD, Kandel ER (1989). 5-Ht modulates protein-synthesis and the expression of specific proteins during long-term facilitation in *Aplysia* sensory neurons. Neuron.

[24] Orban PC, Chapman PF, Brambilla R (1999). Is the Ras-MAPK signalling pathway necessary for long-term memory formation?. Trends Neurosci.

[25] Threadgill R, Bobb K, Ghosh A (1997). Regulation of dendritic growth and remodeling by Rho, Rac, and Cdc42. Neuron.

[26] Sone M, Hoshino M, Suzuki E, Kuroda S, Kaibuchi K, Nakagoshi H (1997). Still life, a protein in synaptic terminals of *Drosophila* homologous to GDP-GTP exchangers. Science.

[27] Kawasaki H, Sprihtgett GM, Toki S, Canales JJ, Harlan P, Blumenstiel JP (1998). A Rap guanine nucleotide exchange factor enriched highly in the basal ganglia. Proc Natl Acad Sci U S A.

[28] Xia S, Miyashita T, Fu T-F, Lin W-Y, Wu C-L, Pyzocha L (2005). NMDA receptors mediate olfactory learning and memory in *Drosophila*. Curr Biol.

[29] Riedel G, Platt B, Micheau J (2003). Glutamate receptor function in learning and memory. Behav Brain Res.

[30] Tubon TC, Zhang J, Friedman EL, Jin H, Gonzales ED, Zhou H (2013). dCREB2-mediated enhancement of memory formation. J Neurosci.

[31] Iyer SC, Iyer EPR, Meduri R, Rubaharan M, Kuntimaddi A, Karamsetty M (2013). Cut, via CrebA, transcriptionally regulates the COPII secretory pathway to direct dendrite development in *Drosophila*. J Cell Sci.

[32] Zhang JB, Yin JCP, Wesley CS (2013). From *Drosophila* development to adult: clues to Notch function in long-term memory. Front Cell Neurosci.

[33] Dubnau J, Chiang AS, Grady L, Barditch J, Gossweiler S, McNeil J (2003). The staufen/pumilio pathway is involved in *Drosophila* long-term memory. Curr Biol.

[34] Shuai YC, Lu BY, Hu Y, Wang LZ, Sun K, Zhong Y (2010). Forgetting is regulated through Rac activity in *Drosophila*. Cell.

[35] Yamada K, Nabeshima T (2003). Brain-derived neurotrophic Factor/TrkB signaling in memory processes. J Pharmacol Sci.

[36] Chan CC, Scoggin S, Wang D, Cherry S, Dembo T, Greenberg B (2011). Systematic discovery of Rab GTPases with synaptic functions in *Drosophila*. Curr Biol.

[37] Abel T, Kandel E (1998). Positive and negative regulatory mechanisms that mediate long-term memory storage. Brain Res Rev.

[38] Pravosudov VV, Roth TC, Forister ML, Ladage LD, Kramer R, Schilkey F (2013). Differential hippocampal gene expression is associated with climate-related natural variation in memory and the hippocampus in food-caching chickadees. Mol Ecol.

[39] Armbrecht HJ, Siddiqui AM, Green M, Farr SA, Kumar VB, Banks WA (2014). SAMP8 mice have altered hippocampal gene expression in long term potentiation, phosphatidylinositol signaling, and endocytosis pathways. Neurobiol Aging.

[40] Klappenbach M, Kaczer L, Locatelli F (2013). Dopamine interferes with appetitive long-term memory formation in honey bees. Neurobiol Learn Mem.

[41] Berry JA, Cervantes-Sandoval I, Nicholas EP, Davis RL (2012). Dopamine is required for learning and forgetting in *Drosophila*. Neuron.

[42] Zhang Y, Shan B, Boyle M, Liu J, Liao L, Xu T (2014). Brain proteome changes induced by olfactory learning in *Drosophila*. J Proteome Res.

[43] Pannebakker BA, Trivedi U, Blaxter MA, Watt R, Shuker DM (2013). The transcriptomic basis of oviposition behaviour in the parasitoid wasp *Nasonia vitripennis*. PLoS One.

[44] Lipscombe D (2005). Neuronal proteins custom designed by alternative splicing. Curr Opin Neurobiol.

[45] Hermey G, Mahlke C, Gutzmann JJ, Schreiber J, Bluthgen N, Kuhl D (2013). Genome-wide profiling of the activity-dependent hippocampal transcriptome. PLoS One.

[46] Banerjee P, Schoenfeld BP, Bell AJ, Choi CH, Bradley MP, Hinchey P (2010). Short- and long-term memory are modulated by multiple isoforms of the fragile X mental retardation protein. J Neurosci.

[47] Mattick JS (2003). Challenging the dogma: the hidden layer of non-protein-coding RNAs in complex organisms. Bioessays.

[48] Pelechano V, Steinmetz LM (2013). Gene regulation by antisense transcription. Nat Rev Genet.

[49] Levenson JM, Sweatt JD (2006). Epigenetic mechanisms: a common theme in vertebrate and invertebrate memory formation. Cell Mol Life Sci.

[50] Lynch JA, Desplan C (2006). A method for parental RNA interference in the wasp *Nasonia vitripennis*. Nat Protoc.

[51] Schroeder A, Mueller O, Stocker S, Salowsky R, Leiber M, Gassmann M (2006). The RIN: an RNA integrity number for assigning integrity values to RNA measurements. BMC Mol Biol.

[52] Grabherr MG, Haas BJ, Yassour M, Levin JZ, Thompson DA, Amit I (2011). Full-length transcriptome assembly from RNA-Seq data without a reference genome. Nat Biotechnol.

[53] GO terms linked to the Nvit 2.0 proteome [http://www.hymenopteragenome.org/nasonia/?q=evidential_gene_data]

