# Supplementary material for: Learning-induced gene expression in the heads of two Nasonia species that differ in long-term memory formation
Source: BMC Genomics. 2015 Mar 10;16(1):162. doi: 10.1186/s12864-015-1355-1 (PMC4440501; doi:10.1186/s12864-015-1355-1)
Supplement: Additional file 1: Figure S1. — Multi-dimension scaling plots of the biological coefficients of variation. Figure S2. Distribution of DE transcripts. Table S1. transcriptome analysis statistics. Table S2. Comparison of naïve gene expression levels in the heads of N. vitripennis and N. giraulti. Table S3. Enriched GO terms in genes that differ in expression levels between naïve N. vitripennis and N. giraulti. Table S4. Enriched GO terms in differentially expressed protein-coding transcripts. Table S5. DE transcripts involved in signalling in N. vitripennis. Table S6. Differential expression of known memory genes after conditioning. Table S7. Differentially expressed genes with an opposed expression pattern after conditioning in N. vitripennis and N. giraulti. Table S8. Enriched GO terms in antisense transcripts. Table S9. Antisense transcripts. [file 12864_2015_1355_MOESM1_ESM.pdf]

Learning-induced gene expression in the heads of two *Nasonia* species that differ in long-term memory formation

Katja M. Hoedjes, Hans M. Smid, Elio G. W. M. Schijlen, Louise E. M. Vet, Joke J. F. A. van Vugt

--Figure S1--

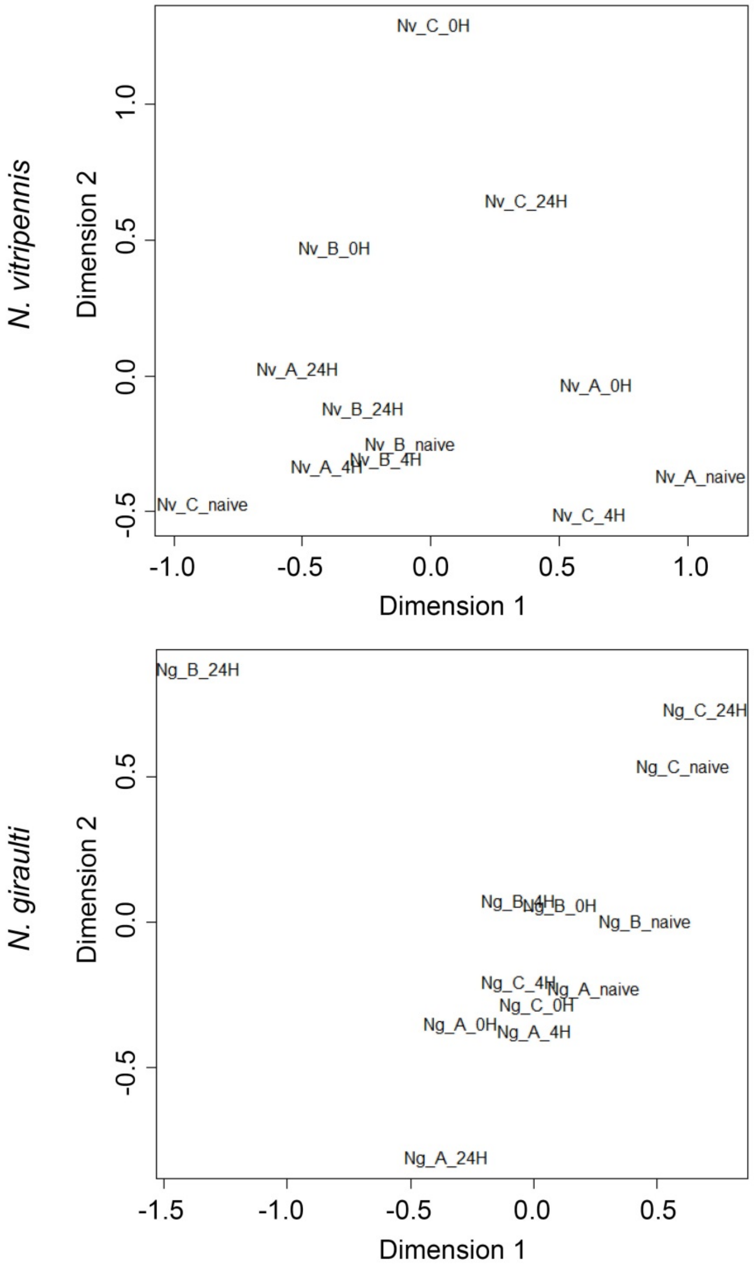

Multi-dimension scaling plots of the biological coefficients of variation. Variation between experimental treatments (0h, 4h, 24h and naïve) and replicates (A, B, C) was analyzed for *N. vitripennis* (top) and *N. giraulti* separately (bottom). The scaling plots reveal that the gene expression data was not affected by a replicate effect. Also, no clear clustering based on experimental treatment is visible.

--Figure S2--

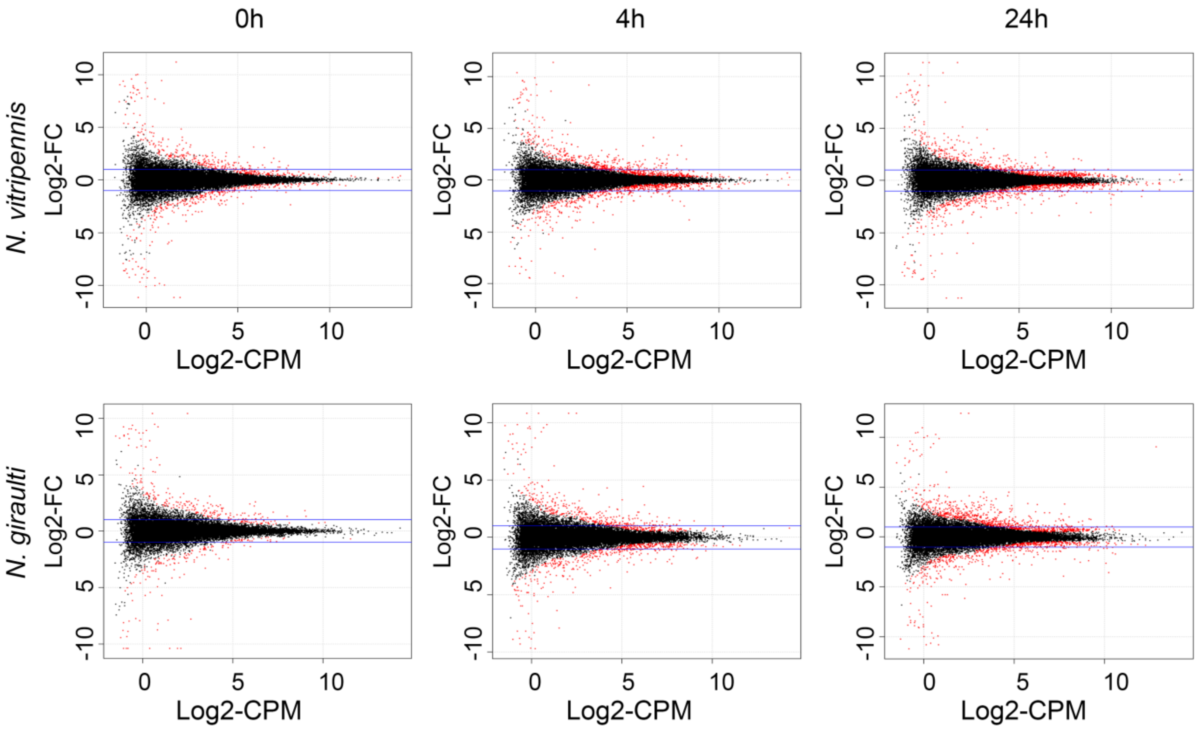

Distribution of DE transcripts. The log2-fold change (FC) and log2-count-per-million (CPM) are plotted for each transcript at the indicated time points. Transcripts that are significantly DE compared to naïve expression are shown in red, whereas transcripts that are not are shown in black.

--Table S1--

Transcriptome analysis statistics. The numbers of reads before and after quality filtering and adapter trimming are shown, together with statistics of the *de novo* transcriptome assemblies for both *N. vitripennis* and *N. giraulti*. These statistics include the number of genes and transcripts both before and after filtering (> 1 cpm for at least 3 samples). The definition of genes and transcripts has been described in the materials and methods section. The number of genes with either one or multiple splice variants (transcripts) is also given. The transcriptome size in bp (i.e. the combined length of the genes), the N50 (the number of transcripts with the largest sizes which together make up for half the transcriptome) and maximum transcript length were determined from the filtered transcripts.

	N. vitripennis	*N. giraulti*
Raw reads	294,289,458	294,210,825
Filtered reads	218,100,037	220,260,984
Unfiltered genes	92,097	82,351
Unfiltered transcripts	139,448	129,060
Filtered genes	15,789	15,453
Single splice variant	11,574	11,274
Multiple splice variants	4,215	4,179
Filtered transcripts	30,223	29,641
Transcriptome size (bp)	33,232,483	32,353,139
N50	4,949	5,019
Maximum transcript length	48,825	49,445

--Table S2--

Comparison of naïve gene expression levels in the heads of *N. vitripennis* and *N. giraulti*. Differences in naïve gene expression levels between the two species were determined by comparing differential gene expression of naïve *N. giraulti* to naïve *N. vitripennis* expression. As two separate transcriptomes had been made for the two species, not all transcripts could be compared to each other, but only the transcripts that were present in both species, as determined by a best-hit blast to the Nvit2.0 proteome. Transcripts of the same transcriptome that had a hit to the same Nvit2.0 protein (for example as a result of splice variation) were removed, as it is uncertain which of these transcripts could be compared with transcript(s) with a best blast hit to the same Nvit2.0 protein of the other species. Differential expression analysis, using EdgeR with GLM trended dispersion with Pearson correlation (as described in the Methods section), was done using naïve *N. vitripennis* as a reference. Consequently, a total of 4275 transcripts, of the 9412 overlapping transcripts, could be compared amongst both species. Of these transcripts 957 were upregulated in *N. giraulti* compared to *N. vitripennis*, 993 were downregulated. The table is shown in Additional_file_2.

--Table S3--

Enriched GO terms in genes that differ in expression levels between naïve *N. vitripennis* and *N. giraulti*. GO enrichment analyses were done using Blast2go (Fisher's exact test, $P < 0.05$) for genes that had a higher or lower expression level in naïve *N. giraulti* as compared to naïve *N. vitripennis* using the set of genes that could be compared between the two species as a reference. Generic GOSlim categories and the Blast2go option 'reduce to most specific terms' were used to limit the number of GO term categories. The enriched GO terms (GO ID and Term), the category of the GO term (C = cellular component, F = molecular function, P = biological process), their P-value and the number of genes in the test-set and the reference-set are given.

Upregulated terms in naïve *N. giraulti* compared to naïve *N. vitripennis*

GO ID	Category	Term	P-Value	#Test	#Ref
GO:0005840	C	ribosome	2.34E-09	45	9
GO:0005198	F	structural molecule activity	6.72E-08	48	14
GO:0006412	P	translation	1.60E-02	45	34

Downregulated terms in naïve *N. giraulti* compared to naïve *N. vitripennis*

GO ID	Category	Term	P-Value	#Test	#Ref
GO:0007267	P	cell-cell signaling	7.07E-03	22	11
GO:0040007	P	growth	1.95E-02	15	7
GO:0008135	F	translation factor activity, nucleic acid binding	2.52E-02	12	5
GO:0006139	P	nucleobase-containing compound metabolic process	3.13E-02	90	87
GO:0044767	P	single-organism developmental process	4.97E-02	85	84

--Table S4--

Enriched GO terms in differentially expressed protein-coding transcripts. GO enrichment analyses were done using Blast2go (Fisher's exact test, $P < 0.05$) for differentially expressed genes that are unique for *N. vitripennis*, unique for *N. giraulti*, and for genes that are differentially expressed at different time-points after conditioning of both species. Generic GOSlim categories and the Blast2go option 'reduce to most specific terms' were used to limit the number of GO term categories. The enriched GO terms (GO ID and Term), the category of the GO term (C = cellular component, F = molecular function, P = biological process), their P-value and the number of genes in the test-set and the reference-set are given.

Differentially expressed transcripts unique for *N. vitripennis*

GO ID	Category	Term	*P*-Value	#Test	#Ref
GO:0003824	F	catalytic activity	3,62E-04	280	2071
GO:0005509	F	calcium ion binding	2,29E-03	21	85
GO:0009719	P	response to endogenous stimulus	4,07E-03	11	33
GO:0008289	F	lipid binding	2,25E-02	12	50
GO:0005102	F	receptor binding	2,25E-02	12	50
GO:0000003	P	reproduction	3,57E-02	43	275
GO:0006629	P	lipid metabolic process	3,70E-02	19	101
GO:0019748	P	secondary metabolic process	4,79E-02	5	15

Differentially expressed transcripts unique for *N. giraulti*

GO ID	Category	Term	*P*-Value	#Test	#Ref
GO:0000166	F	nucleotide binding	1,55E-05	109	731
GO:0006259	P	DNA metabolic process	2,59E-04	25	108
GO:0007049	P	cell cycle	3,81E-04	45	257
GO:0009058	P	biosynthetic process	5,20E-03	70	512
GO:0007010	P	cytoskeleton organization	1,04E-02	33	211
GO:0008135	F	translation factor activity, nucleic acid binding	1,07E-02	13	59
GO:0019538	P	protein metabolic process	1,89E-02	96	785
GO:0030234	F	enzyme regulator activity	3,17E-02	20	124
GO:0003774	F	motor activity	3,32E-02	7	28
GO:0030246	F	carbohydrate binding	3,48E-02	6	22
GO:0016301	F	kinase activity	4,91E-02	45	348

***N. vitripennis* 0H after conditioning - Upregulated**

GO ID	Category	Term	*P*-Value	#Test	#Ref
GO:0005509	F	calcium ion binding	1,32E-03	10	96
GO:0007165	P	signal transduction	5,32E-03	30	584
GO:0005215	F	transporter activity	2,57E-02	18	344

GO:0005811	C	lipid particle	2,74E-02	6	70
GO:0009719	P	response to endogenous stimulus	4,37E-02	4	40

***N. vitripennis* 0H after conditioning - Downregulated**

GO ID	Category	Term	*P*-Value	#Test	#Ref
GO:0050896	P	response to stimulus	1,84E-02	33	936
GO:0016032	P	viral process	3,74E-02	2	11
GO:0004871	F	signal transducer activity	4,81E-02	5	78

***N. vitripennis* 4H after conditioning - Upregulated**

GO ID	Category	Term	*P*-Value	#Test	#Ref
GO:0005840	C	ribosome	1,27E-14	32	78
GO:0006412	P	translation	2,08E-11	42	186
GO:0005198	F	structural molecule activity	1,90E-09	33	142
GO:0003723	F	RNA binding	1,89E-04	28	200
GO:0005730	C	nucleolus	2,49E-03	13	77
GO:0000003	P	reproduction	4,36E-03	31	287
GO:0008283	P	cell proliferation	1,37E-02	13	97
GO:0009719	P	response to endogenous stimulus	1,46E-02	7	37
GO:0005215	F	transporter activity	4,10E-02	30	332

***N. vitripennis* 4H after conditioning - Downregulated**

GO ID	Category	Term	*P*-Value	#Test	#Ref
GO:0005811	C	lipid particle	7,62E-04	10	66
GO:0016787	F	hydrolase activity	1,30E-03	54	866
GO:0005975	P	carbohydrate metabolic process	2,24E-03	13	120
GO:0005764	C	lysosome	1,98E-02	3	12
GO:0019748	P	secondary metabolic process	4,29E-02	3	17
GO:0006629	P	lipid metabolic process	4,84E-02	9	111

***N. vitripennis* 24H after conditioning - Upregulated**

GO ID	Category	Term	*P*-Value	#Test	#Ref
GO:0005840	C	ribosome	1,51E-50	63	47
GO:0005198	F	structural molecule activity	6,01E-35	63	112
GO:0006412	P	translation	3,64E-29	65	163
GO:0005811	C	lipid particle	2,37E-07	18	58
GO:0005829	C	cytosol	5,32E-03	17	130
GO:0003723	F	RNA binding	7,22E-03	23	205

***N. vitripennis* 24H after conditioning - Downregulated**

GO ID	Category	Term	*P*-Value	#Test	#Ref
GO:0005509	F	calcium ion binding	6,26E-06	18	88
GO:0003824	F	catalytic activity	2,03E-05	155	2196
GO:0019748	P	secondary metabolic process	3,98E-05	7	13
GO:0005975	P	carbohydrate metabolic process	4,41E-04	17	116
GO:0005811	C	lipid particle	1,65E-03	11	65

GO:0016209	F	antioxidant activity	2,49E-03	4	8
GO:0006629	P	lipid metabolic process	3,35E-03	14	106
GO:0008289	F	lipid binding	4,21E-03	9	53
GO:0009719	P	response to endogenous stimulus	6,77E-03	7	37
GO:0019825	F	oxygen binding	7,73E-03	2	1
GO:0009056	P	catabolic process	1,06E-02	30	346
GO:0008092	F	cytoskeletal protein binding	1,81E-02	14	132
GO:0006091	P	generation of precursor metabolites and energy	2,57E-02	10	86

***N. giraulti* 0H after conditioning - Upregulated**

GO ID	Category	Term	*P*-Value	#Test	#Ref
GO:0003700	F	sequence-specific DNA binding transcription factor activity	4,50E-04	11	166
GO:0003677	F	DNA binding	5,12E-04	16	322
GO:0000166	F	nucleotide binding	5,80E-03	26	814
GO:0050789	P	regulation of biological process	6,89E-03	38	1362
GO:0008219	P	cell death	2,16E-02	8	175
GO:0009653	P	anatomical structure morphogenesis	3,73E-02	18	597
GO:0005215	F	transporter activity	4,77E-02	12	362

***N. giraulti* 0H after conditioning - Downregulated**

GO ID	Category	Term	*P*-Value	#Test	#Ref
GO:0003779	F	actin binding	2,34E-02	4	84
GO:0044699	P	single-organism process	4,89E-02	31	1897

***N. giraulti* 4H after conditioning - Upregulated**

GO ID	Category	Term	*P*-Value	#Test	#Ref
GO:0005730	C	nucleolus	1,29E-06	16	80
GO:0008135	F	translation factor activity, nucleic acid binding	1,40E-04	11	61
GO:0000166	F	nucleotide binding	1,57E-04	55	785
GO:0006412	P	translation	3,24E-04	21	205
GO:0006139	P	nucleobase-containing compound metabolic process	5,31E-03	37	556

***N. giraulti* 4H after conditioning - Downregulated**

GO ID	Category	Term	*P*-Value	#Test	#Ref
GO:0005975	P	carbohydrate metabolic process	7,16E-06	16	117
GO:0006629	P	lipid metabolic process	1,58E-02	9	108
GO:0006091	P	generation of precursor metabolites and energy	2,11E-02	8	95
GO:0019748	P	secondary metabolic process	3,13E-02	3	18
GO:0005811	C	lipid particle	4,28E-02	6	71
GO:0016787	F	hydrolase activity	4,74E-02	40	889

***N. giraulti* 24H after conditioning - Upregulated**

GO ID		Term	*P*-Value	#Test	#Ref
GO:0005840	C	ribosome	4,44E-47	65	45
GO:0006412	P	translation	1,74E-42	86	140
GO:0005198	F	structural molecule activity	6,01E-33	68	113
GO:0007049	P	cell cycle	7,24E-07	46	256
GO:0008135	F	translation factor activity, nucleic acid binding	1,01E-05	17	55
GO:0005829	C	cytosol	1,36E-05	26	120
GO:0005811	C	lipid particle	9,83E-05	16	61
GO:0000166	F	nucleotide binding	4,63E-03	80	760
GO:0005694	C	chromosome	8,43E-03	17	108
GO:0007010	P	cytoskeleton organization	9,75E-03	28	216
GO:0006259	P	DNA metabolic process	1,52E-02	17	116
GO:0009055	F	electron carrier activity	2,65E-02	5	19
GO:0005730	C	nucleolus	4,40E-02	12	84

***N. giraulti* 24H after conditioning - Downregulated**

GO ID	Category	Term	*P*-Value	#Test	#Ref
GO:0005509	F	calcium ion binding	2,07E-04	13	86
GO:0005975	P	carbohydrate metabolic process	3,78E-04	15	118
GO:0003774	F	motor activity	2,89E-03	6	29
GO:0006629	P	lipid metabolic process	9,15E-03	11	106
GO:0005811	C	lipid particle	1,42E-02	8	69
GO:0005215	F	transporter activity	2,16E-02	24	350
GO:0052689	F	carboxylic ester hydrolase activity	3,16E-02	2	5
GO:0019725	P	cellular homeostasis	3,26E-02	6	52
GO:0008037	P	cell recognition	3,49E-02	5	39

--Table S5--

DE transcripts involved in signalling in *N. vitripennis*. Presented here are the differentially expressed transcripts in *N. vitripennis* immediately after conditioning, which were clustered in the enriched GO terms 'signal transduction' (GO:0007165), 'response to endogenous stimulus' (GO:0009719) and 'response to stimulus' (GO:0050896). The log2-fold change (FC), log2-count per million (cpm) and the level of significance of the transcripts are given. In addition, the DE pattern of each transcript is shown for *N. vitripennis* (*NV*) and *N. giraulti* (*NG*); ↓ = downregulated, ↑ = upregulated, n.a. = transcript not available in the transcriptome. The transcripts were ordered based on their absolute fold change.

OGS2.0 ID	Transcript ID	Gene description	log2-FC	log2-cpm	P-value	DE pattern *NV*	DE pattern *NG*
Nasvi2EG013237t1	comp39228_c0_seq3	Still life	-10,14	0,02	3,37E-08	↓0h,24h	not DE
Nasvi2EG008626t2	comp483118_c0_seq1	Unknown	8,20	-0,35	9,96E-05	↑0h,4h,24h	not DE
Nasvi2EG013237t2	comp29402_c0_seq1	Still life	7,71	-0,77	2,30E-04	↑0h,4h,24h	not DE
Nasvi2EG001811t1	comp38721_c1_seq2	Unknown	-5,90	0,36	4,58E-07	↓0h	n.a
Nasvi2EG023096t1	comp34611_c0_seq2	Lysine-specific histone demethylase 1A	5,43	-0,47	6,12E-05	↑0h	not DE
Nasvi2EG017531t1	comp38970_c2_seq11	TGF-beta receptor type-1	-4,50	0,69	1,19E-05	↓0h	not DE
Nasvi2EG037341t1	comp37043_c0_seq6	Ras-related protein Rab-32	4,30	-0,16	4,54E-04	↑0h	↓4h,24h
Nasvi2EG019393t1	comp39881_c3_seq1	Glutamate receptor subunit 1	4,20	-0,44	5,82E-04	↑0h	not DE
Nasvi2EG014089t3	comp37425_c0_seq2	TBC1 domain family member CG11727	4,01	1,99	1,27E-08	↑0h,4h,24h	n.a
Nasvi2EG003595t2	comp27330_c0_seq1	SLIT-ROBO Rho GTPase-activating protein 1	3,92	0,92	1,07E-06	↑0h,4h,24h	not DE
Nasvi2EG009337t1	comp36228_c0_seq7	Tyrosine-protein kinase receptor	3,82	1,21	3,21E-07	↑0h	not DE
Nasvi2EG004424t1	comp39301_c4_seq16	Dystrophin, isoforms A/C/F/G/H	-3,81	-0,06	5,77E-04	↓0h	n.a
Nasvi2EG023114t1	comp39910_c1_seq23	Phosphatidylinositol 3-kinase catalytic subunit type 3	3,80	-0,13	3,27E-04	↑0h	↓0h
Nasvi2EG003595t2	comp27330_c0_seq4	SLIT-ROBO Rho GTPase-activating protein 1	3,72	1,99	4,72E-08	↑0h,4h,24h	not DE
Nasvi2EG009337t1	comp36228_c0_seq6	Tyrosine-protein kinase receptor	3,68	0,31	1,41E-04	↑0h	not DE
Nasvi2EG008869t3	comp39661_c11_seq7	Unknown	-3,46	0,13	3,28E-04	↓0h	n.a

Nasvi2EG007191t1	comp13537_c0_seq1	Death related ced-3/Nedd2 protein	-3,39	0,46	8,47E-05	↓0h	not DE
Nasvi2EG006407t3	comp35269_c1_seq1	Tyrosine-protein kinase Src64B	-3,39	0,16	8,61E-04	↓0h	not DE
Nasvi2EG013660t1	comp33709_c0_seq3	Biotin--protein ligase	-2,89	0,50	4,04E-04	↓0h	↓4h
Nasvi2EG014089t3	comp37425_c0_seq17	TBC1 domain family member CG11727	2,86	1,65	1,75E-05	↑0h	n.a
Nasvi2EG004379t4	comp37393_c0_seq4	E3 ubiquitin-protein ligase UBR2	-2,73	1,16	9,18E-05	↓0h	not DE
Nasvi2EG014516t1	comp39309_c2_seq19	Ras-related protein Rab-6A	-2,52	2,39	6,00E-06	↓0h	not DE
Nasvi2EG002305t1	comp32573_c0_seq3	GTP-binding protein Di-Ras2	2,51	0,72	3,76E-04	↑0h	not DE
Nasvi2EG015011t1	comp37930_c2_seq4	Parathyroid hormone-related peptide receptor	-2,42	1,23	1,47E-04	↓0h	↓24h
Nasvi2EG014393t1	comp43869_c0_seq1	Unknown	2,35	1,96	5,24E-05	↑0h,4h	not DE
Nasvi2EG015580t1	comp35764_c0_seq4	Armadillo segment polarity protein	-2,30	5,31	5,21E-28	↓0h,4h,24h	↓24h
Nasvi2EG005268t1	comp34705_c0_seq5	Crumbs protein	2,27	3,11	6,31E-08	↑0h,4h,24h	not DE
Nasvi2EG011643t3	comp26279_c0_seq1	DNA repair endonuclease XPF	2,26	1,30	4,00E-04	↑0h	n.a
Nasvi2EG005229t1	comp27827_c0_seq2	Signal transducing adapter molecule 1	-2,25	2,75	8,24E-06	↓0h	not DE
Nasvi2EG019874t3	comp38852_c1_seq15	Triple functional domain protein	2,25	1,87	1,17E-04	↑0h	not DE
Nasvi2EG015607t1	comp36072_c0_seq2	Deoxyribodipyrimidine photo-lyase	-2,16	1,86	3,48E-04	↓0h	not DE
Nasvi2EG010744t1	comp39149_c0_seq12	Metabotropic glutamate receptor (mangetout)	-2,14	1,04	1,12E-03	↓0h,4h,24h	not DE
Nasvi2EG000868t1	comp38978_c0_seq7	Tachykinin peptides receptor 99D-like	2,01	1,21	9,61E-04	↑0h	↑4h,24h
Nasvi2EG022273t2	comp36648_c0_seq8	GTP-binding protein Rheb homolog	-1,97	4,08	8,19E-10	↓0h	not DE
Nasvi2EG014089t3	comp37425_c0_seq5	TBC1 domain family member CG11727	-1,95	4,41	5,77E-10	↓0h,24h	n.a
Nasvi2EG015809t1	comp33742_c0_seq1	Innexin inx1	1,87	3,41	5,32E-07	↑0h,4h	not DE
Nasvi2EG009786t4	comp38864_c1_seq5	Guanine nucleotide-releasing factor 2	-1,83	3,75	1,86E-07	↓0h	not DE
Nasvi2EG008107t4	comp27284_c0_seq3	OTU domain-containing protein 7B, putative	1,78	3,27	2,37E-06	↑0h,4h	not DE
Nasvi2EG002499t2	comp39261_c0_seq105	Retinal degeneration B protein	1,72	2,72	1,17E-03	↑0h,4h,24h	↓24h
Nasvi2EG007375t3	comp33212_c0_seq3	E3 ubiquitin-protein ligase suppressor of deltex	-1,65	2,98	1,31E-04	↓0h	not DE
Nasvi2EG015580t1	comp35764_c0_seq5	Armadillo segment polarity protein	1,62	4,88	1,38E-12	↑0h,4h,24h	↓24h
Nasvi2EG009786t4	comp38864_c1_seq7	Guanine nucleotide-releasing factor 2	-1,58	3,26	9,43E-05	↓0h	not DE
Nasvi2EG003715t2	comp37447_c0_seq28	Afadin	-1,58	4,66	4,92E-09	↓0h	not DE
Nasvi2EG009786t4	comp38864_c1_seq6	Guanine nucleotide-releasing factor 2	1,48	2,92	6,04E-04	↑0h,4h,24h	not DE
Nasvi2EG008626t2	comp38080_c0_seq26	Unknown	1,43	4,34	6,29E-07	↑0h ↓24h	not DE
Nasvi2EG001535t1	comp34948_c0_seq2	Serine/threonine-protein kinase SNF1 kinase 2	1,40	2,97	7,75E-04	↑0h,24h	not DE

Nasvi2EG037341t1	comp37043_c0_seq1	Ras-related protein Rab-32	-1,39	3,46	4,55E-05	↓0h,4h,24h	↓4h,24h
Nasvi2EG009786t4	comp38864_c1_seq3	Guanine nucleotide-releasing factor 2	1,20	3,45	5,82E-04	↑0h,4h,24h	not DE
Nasvi2EG003084t2	comp26839_c0_seq1	Reticulon-1	-1,20	7,63	1,33E-24	↓0h,4h,24h	↓0h,24h
Nasvi2EG037152t1	comp31826_c2_seq2	Ecdysone-induced protein 75B	1,17	7,20	1,75E-21	↑0h,4h,24h	↑0h,4h,24h
Nasvi2EG006681t8	comp39113_c5_seq5	Arginine-glutamic acid dipeptide repeats protein	-1,14	4,12	2,65E-04	↓0h	n.a
Nasvi2EG025129t1	comp34836_c0_seq1	B-cell lymphoma 3-encoded protein	-1,13	4,29	8,02E-05	↓0h	↑0h,4h
Nasvi2EG007441t6	comp38606_c0_seq20	1-phosphatidylinositol-4,5-bisphosphate phosphodiesterase	-1,12	4,56	1,64E-05	↓0h	↑24h
Nasvi2EG011878t2	comp35527_c5_seq2	Roadkill, protein	0,89	7,21	3,60E-13	↑0h,4h	↑0h
Nasvi2EG005342t1	comp42369_c0_seq1	Raf protein serine/threonine-protein kinase dRAF-1	0,85	5,78	1,84E-08	↑0h	↑0h
Nasvi2EG002993t3	comp39642_c22_seq8	Serine/threonine-protein phosphatase 2A regulatory subunit	0,84	5,68	8,05E-08	↑0h	not DE
Nasvi2EG010931t1	comp14073_c0_seq2	Mob as tumor suppressor	-0,81	5,62	1,07E-06	↓0h ↑4h,24h	↓0h,4h
Nasvi2EG007441t4	comp38606_c0_seq1	1-phosphatidylinositol-4,5-bisphosphate phosphodiesterase	-0,80	5,04	1,18E-04	↓0h	not DE
Nasvi2EG013237t2	comp39228_c0_seq8	Still life, protein	-0,73	5,07	3,44E-04	↓0h	not DE
Nasvi2EG008393t1	comp40145_c0_seq1	Antimicrobial peptide Def1-1	-0,60	7,57	2,01E-07	↓0h	↑4h,24h
Nasvi2EG013710t2	comp37252_c0_seq1	ADIPOR receptor CG5315-like, putative	0,58	6,91	7,06E-06	↑0h,4h,24h	not DE
Nasvi2EG002888t3	comp13915_c1_seq1	Spinster protein, putative	-0,57	5,46	6,96E-04	↓0h	↓4h
Nasvi2EG014068t1	comp41689_c0_seq1	Transcription factor AP-1	0,57	5,86	1,39E-04	↑0h	↑24h
Nasvi2EG008392t1	comp40158_c0_seq1	Antimicrobial peptide Def1-1	-0,56	5,97	1,45E-04	↓0h,24h	↑24h
Nasvi2EG010166t1	comp14378_c0_seq1	Transforming protein Ski	0,53	6,74	5,05E-05	↑0h	not DE
Nasvi2EG009605t2	comp32432_c0_seq1	Tyrosine-protein kinase Src42A, putative	-0,51	5,82	7,88E-04	↓0h	not DE
Nasvi2EG007947t2	comp39607_c4_seq2	Guanine nucleotide-binding protein G(q) subunit alpha	0,51	6,04	3,73E-04	↑0h	not DE
Nasvi2EG033203t1	comp13782_c0_seq1	cGMP-dependent 3',5'-cyclic phosphodiesterase	0,50	5,96	6,74E-04	↑0h	↑0h
Nasvi2EG003084t2	comp26839_c0_seq2	Reticulon-1	0,42	8,42	3,79E-05	↑0h	↓0h,24h
Nasvi2EG006840t1	comp40159_c0_seq1	Nuclear protein 1	-0,38	10,11	1,01E-06	↓0h,4h	not DE
Nasvi2EG003943t4	comp27087_c0_seq1	Myosin heavy chain, muscle	0,27	11,41	7,00E-06	↑0h ↓24h	↓24h

--Table S6--

Differential expression of known memory genes after conditioning. (a) The gene expression patterns of 78 genes that are known from literature to be involved in (long-term) memory formation were analysed for both species. The number of transcripts that was observed in the transcriptome is given for each gene. Also, the differential expression pattern after conditioning is given, compared to naïve expression levels (not DE = not differentially expressed, n.a. = not available in the transcriptome, ↑ = upregulated, ↓ = downregulated). (b) 37 genes were differentially expressed in *N. vitripennis* (NV) and/or *N. giraulti* (NG) after conditioning. The transcript IDs are given for each transcript that is DE (e.g. 'comp35553_c1_seq4'), but not for other transcripts of the same gene. When none of the transcripts of a gene are DE, the name of the gene is given (e.g. 'comp28144_c1'). For each DE transcript, the log2-count per million (cpm), log2-fold change (FC), and the level of significance (*P*) are given for the time points at which the transcripts are differentially expressed.

a. Gene expression patterns of 78 known memory genes

Gene/ transgene	Gene description	Reference	OGS2.0 ID	# isoforms *NV*	# isoforms *NG*	DE pattern *NV*	DE pattern *NG*
**Transcription factors and coactivators**							
CBP/p300	CREB binding protein/p300	Alberini 2009	Nasvi2EG004485	25	2	↓ 0h	not DE
C/EBP	CCAAT-enhancer binding protein	Alberini 2009	Nasvi2EG010468	2	2	↑ 0h	↑ 0h
CRTC	CREB-regulated transcription coactivator	Hirano *et al.* 2013	Nasvi2EG020434	10	15	not DE	not DE
dCREB2	cAMP response element binding protein B	Alberini 2009	Nasvi2EG004930	11	9	not DE	↓ 4h,24h
dCREBA	cAMP response element binding protein A	Iyer *et al.* 2013	Nasvi2EG009270	2	1	not DE	↑ 0h
Nalyot	Adh transcription factor 1	Keene & Waddell 2007	Nasvi2EG008127	1	1	not DE	not DE
Stat92E	STAT transcription factor	Copf *et al.* 2011	Nasvi2EG007751	2	2	not DE	not DE
Su(H)	Suppressor of Hairless transcription coactivator	Song *et al.* 2009	Nasvi2EG019939	3	2	↑ 0h,4h	not DE
			Nasvi2EG020439	4	1	↑ 0h	not DE
**Translation factors**							
eIF-5C	elongation initiation factor-5C	Keene & Waddell 2007	Nasvi2EG002237	1	1	↑ 4h	↑ 24h

Orb2	cytoplasmic polyadenylation element binding	Pai *et al*. 2013	Nasvi2EG015611	6	5	↑ 4h	not DE
Oskar	translation control	Keene & Waddell 2007	Nasvi2EG013571	1	1	not DE	not DE
Pumilio	RNA binding protein	Keene & Waddell 2007	Nasvi2EG006115	4	4	↓ 4h	not DE
**Protein kinases**							
CAMKI	calcium/calmodulin-dependent protein kinase I	Schmitt *et al*. 2005	Nasvi2EG016557	2	1	↓ 4h	not DE
CAMKII	calcium/calmodulin-dependent protein kinase II	Alberini 2009	Nasvi2EG036901	16	16	not DE	↑ 0h,4h,24h
CASK	CASK ortholog	Malik *et al*. 2013	Nasvi2EG011836	16	24	not DE	not DE
DC0	cAMP-dependent protein kinase 1	Keene & Waddell 2007	Nasvi2EG014242	1	1	not DE	not DE
ERK-A	extracellular signal-related kinase	Alberini 2009	Nasvi2EG002789	1	2	not DE	not DE
Foraging	cGMP dependent protein kinase	Mery *et al*. 2007	Nasvi2EG004831	2	2	not DE	↓ 24h
Hopscotch	Janus tyrosine kinase	Copf *et al*. 2011	Nasvi2EG014300	2	2	↓ 0h	not DE
Linotte	RYK receptor tyrosine kinase	Keene & Waddell 2007	Nasvi2EG007570	2	1	↓ 0h	not DE
p38 MAPK	p38 mitogen activated protein kinase	Alberini 2009	Nasvi2EG000447	8	8	not DE	not DE
PKA3	cAMP-dependent protein kinase 3	da Cruz *et al*. 2008	Nasvi2EG011355	1	1	↑ 4h	↑ 4h
PKA-RI	cAMP dependent protein kinase R1	Keene & Waddell 2007	Nasvi2EG005068	2	2	not DE	not DE
PKA-R2	cAMP-dependent protein kinase R2	Schwaerzel *et al*. 2007	Nasvi2EG013246	11	30	↑ 4h,24h ↓ 4h	↓ 4h
aPKC	atypical protein kinase C	Keene & Waddell 2007	Nasvi2EG010162	5	3	↑ 4h,24h	not DE
PKC 53E	protein C kinase 53E	Thiels *et al*. 2000	Nasvi2EG009095	2	2	not DE	not DE
PKC 98E	protein C kinase 98E	Zisopoulou *et al*. 2013	Nasvi2EG013022	2	2	not DE	not DE
PKC δ	protein C kinase δ	Gallagher *et al*. 2001	Nasvi2EG004720	9	5	↑ 4h ↓ 4h	not DE
Minibrain	tyrosine-phosphorylation-regulated kinase 1A	Tejedor *et al*. 1995	Nasvi2EG003454	3	4	not DE	not DE
Src64B	Src oncogene at 64B, tyrosine kinase	Akalal *et al*. 2011	Nasvi2EG006407	3	3	↓ 0h	not DE
S6KII	ribosomal protein S6 kinase II	Keene & Waddell 2007	Nasvi2EG013142	1	1	not DE	not DE
**Receptors and channels**							
Nmda1	glutamate receptor-associated protein	Xia *et al*. 2005	Nasvi2EG001699	2	2	not DE	↑ 24h
NMDAR1	glutamate receptor subunit 1	Keene & Waddell 2007	Nasvi2EG019393	3	2	↑ 0h	not DE
NMDAR2	glutamate receptor subunit 2	Keene & Waddell 2007	Nasvi2EG012232	6	5	not DE	↓ 4h
Notch	Notch receptor	Keene & Waddell 2007	Nasvi2EG009801	3	5	not DE	not DE
Clumsy	glutamate receptor	Riedel *et al*. 2003	Nasvi2EG010011	12	4	not DE	↑ 0h,4h,24h ↓ 0h,4h,24h
			Nasvi2EG010019	30	5	not DE	↑ 0h,4h,24h
			Nasvi2EG002756	7	28	not DE	↑ 0h,24h

GluCl	glutamate-gated chloride channel	Liu & Wilson 2013	Nasvi2EG003037	4	4	not DE	not DE
GluRIA	glutamate receptor IA	Bevilaqua *et al*. 2005	Nasvi2EG000002	4	4	not DE	not DE
GluRIB	glutamate receptor IB	Bevilaqua *et al*. 2005	Nasvi2EG000002	4	4	not DE	not DE
Damb	dopamine 1-like receptor 2	Keene & Waddell 2007	Nasvi2EG017876	3	10	not DE	not DE
dDA1	dopamine 1-like receptor 1	Keene & Waddell 2007	Nasvi2EG001652	8	4	↓ 4h	↓ 4h
Dop2R	dopamine 2-like receptor	Hearn *et al*. 2002	Nasvi2EG023476	2	1	not DE	not DE
DopEcR	dopamine/ecdysteroid receptor	Ishimoto *et al*. 2013	Nasvi2EG017695	1	1	not DE	not DE
5-HT1A	5-hydroxytryptamine (serotonin) receptor 1A	Johnson *et al*. 2011	Nasvi2EG009236	1	1	not DE	not DE
5-HT1B	5-hydroxytryptamine (serotonin) receptor 1B	Johnson *et al*. 2011	Nasvi2EG009242	1	1	not DE	not DE
5-HT2A	5-hydroxytryptamine (serotonin) receptor 2A	Johnson *et al*. 2011	Nasvi2EG002123	0	0	n.a.	n.a.
5-HT2B	5-hydroxytryptamine (serotonin) receptor 2B	Johnson *et al*. 2011	Nasvi2EG002123	0	0	n.a.	n.a.
5-HT7	5-hydroxytryptamine (serotonin) receptor 7	Johnson *et al*. 2011	Nasvi2EG036914	1	1	not DE	not DE
Oamb	Octopamine receptor in mushroom bodies	Keene & Waddell 2007	Nasvi2EG003445	1	2	not DE	not DE
Octβ2R	octopamine β2 receptor	Burke *et al*. 2012	Nasvi2EG001703	14	15	not DE	not DE
Oct-TyrR	octopamine-tyramine receptor	Nagaya *et al*. 2002	Nasvi2EG001406	1	1	not DE	not DE
TyrR	tyramine receptor	Homberg *et al*. 2013	Nasvi2EG002052	2	1	not DE	not DE
Volado	α-integrin	Keene & Waddell 2007	Nasvi2EG012335	2	1	not DE	not DE
Eag	ether a go-go, potassium channel	Griffith *et al*. 1994	Nasvi2EG005885	18	20	not DE	not DE
Brp	bruchpilot, calcium channel	Knapek *et al*. 2011	Nasvi2EG000461	4	9	not DE	not DE
**Neurotransmitter synthesis**							
Ddc	dopa decarboxylase	Meiser *et al*. 2013	Nasvi2EG002599	1	1	not DE	not DE
Pale	tyrosine 3-monooxygenase	Meiser *et al*. 2013	Nasvi2EG022898	1	1	↑ 4h ↓ 24h	↓ 24h
Tbh	tyramine beta-hydroxylase	Keene & Waddell 2007	Nasvi2EG012708	1	1	not DE	not DE
Tdc1	tyrosine decarboxylase 1	Meiser *et al*. 2013	Nasvi2EG021102	4	18	not DE	↓ 24h
**Phosphatases, phosphodiesterases, proteases**							
Corkscrew	SHP2 phosphatase	Pagani *et al*. 2009	Nasvi2EG012844	2	2	↑ 4h	not DE
Crammer	trans-inhibitor of cathepsins	Keene & Waddell 2007	Nasvi2EG034880	2	1	↓ 4h,24h	↓ 4h
Dunce	cAMP phosphodiesterase	Keene & Waddell 2007	Nasvi2EG011498	10	13	↑ 4h	↓ 4h
Tequila	neurotrypsin	Keene & Waddell 2007	Nasvi2EG010908	5	4	↓ 4h	not DE
**GTPases and GTPase activating proteins**							
G-sα60A	stimulatory G protein	Keene & Waddell 2007	Nasvi2EG010676	2	1	↑ 24h	not DE

NF1	Ras GTPase activating protein	Keene & Waddell 2007	Nasvi2EG011800	4	7	not DE	↑ 24h
Rac1	Rho family GTPase	Shuai *et al.* 2010	Nasvi2EG000948	1	1	not DE	not DE
Radish	Rap GTPase activating protein	Keene & Waddell 2007	Nasvi2EG000376	2	2	not DE	not DE
**Other genes**							
Armitage	RNA helicase (SDE3)	Keene & Waddell 2007	Nasvi2EG003373	4	1	↓ 4h	not DE
Fasciclin 1	neural cell adhesion molecule	Eickhoff & Bicker 2012	Nasvi2EG020485	6	4	not DE	↓ 24h
Fasciclin 2	neural cell adhesion molecule	Keene & Waddell 2007	Nasvi2EG000400	6	6	↑ 0h,4h,24h	not DE
Fasciclin 3	neural cell adhesion molecule	Dubnau *et al.* 2003	Nasvi2EG003598	10	11	↓ 0h	↑ 4h,24h<br>↓ 4h,24h
FMRP	fragile X mental retardation protein	Banerjee *et al.* 2010	Nasvi2EG013697	8	8	↑ 0h	not DE
Latheo	origin recognition complex	Keene & Waddell 2007	Nasvi2EG008683	1	1	not DE	not DE
Leonardo	14-3-3 zeta protein	Keene & Waddell 2007	Nasvi2EG004640	1	1	not DE	not DE
Sarah	calcineurin inhibitor	Keene & Waddell 2007	Nasvi2EG014610	5	5	not DE	not DE
Rutabaga	adenylyl cyclase (type 2)	Keene & Waddell 2007	Nasvi2EG013346	2	5	not DE	not DE
Staufen	mRNA translocation	Keene & Waddell 2007	Nasvi2EG013994	3	2	↓ 0h	not DE
Synapsin	presynaptic vesicle protein	Keene & Waddell 2007	Nasvi2EG017400	4	4	not DE	not DE

b. Information on 37 DE memory genes

	N. vitripennis					*N. giraulti*				
**Gene**	**Transcript ID**	**Timepoint**	**Log2-CPM**	**Log2-FC**	***P***	**Transcript ID**	**Timepoint**	**Log2-cpm**	**Log2-FC**	***P***
aPKC	comp35553_c1_seq4	↑ 4h	3,88	1,07	9,73E-04	comp28144_c1	not DE	n.a.	n.a	n.a.
		↑ 24h	3,88	0,99	2,09E-03					
Armitage	comp36728_c0_seq1	↓ 4h	5,07	-0,78	1,62E-04	comp50069_c0	not DE	n.a.	n.a.	n.a.
C/EBP	comp14353_c1_seq1	↑ 0h	2,23	1,89	4,99E-04	comp37628_c0_seq1	↑ 0h	4,42	1,76	6,46E-09
	comp15880_c0	not DE	n.a.	n.a.	n.a.	comp22968_c1	not DE	n.a.	n.a.	n.a.
CAMKI	comp35899_c3_seq1	↓ 4h	5,24	-0,62	8,17E-04	comp31541_c2	not DE	n.a.	n.a.	n.a.
CAMKII	comp38849_c3	not DE	n.a.	n.a.	n.a.	comp34936_c1_seq35	↑ 0h	-0,78	7,56	4,91E-04
							↑ 4h	-0,78	9,81	1,09E-06
							↑ 24h	-0,78	7,62	3,86E-04
CBP/p300	comp39271_c0_seq35	↓ 0h	0,80	-2,60	2,14E-04	comp32931_c2	not DE	n.a.	n.a.	n.a.
Clumsy	comp37853_c2	not DE	n.a.	n.a.	n.a.	comp35273_c1_seq12 (1)	↓ 0h	3,39	-1,67	1,71E-05
	comp40094_c0	not DE	n.a.	n.a.	n.a.		↓ 4h	3,39	-1,73	1,36E-05

	comp38794_c0	not DE	n.a.	n.a.	n.a.		↓ 24h	3,39	-1,90	1,30E-06
	comp39218_c1	not DE	n.a.	n.a.	n.a.	comp35273_c1_seq28 (2)	↑ 0h	4,02	1,63	4,58E-06
							↑ 4h	4,02	1,86	3,28E-07
							↑ 24h	4,02	1,81	1,40E-06
						comp35273_c1_seq37 (3)	↑ 0h	2,75	1,89	2,00E-04
							↑ 24h	2,75	1,77	4,97E-04
						comp33862_c0	not DE	n.a.	n.a.	n.a.
						comp33955_c0	not DE	n.a.	n.a.	n.a.
Corkscrew	comp32621_c0_seq1	↑ 4h	7,17	0,49	5,03E-05	comp27751_c0	not DE	n.a.	n.a.	n.a.
Crammer	comp32552_c0_seq1	↓ 4h	8,44	-0,81	2,09E-15	comp21791_c0_seq1	↓ 4h	7,15	-0,71	1,48E-05
		↓ 24h	8,44	-0,57	1,72E-08					
dCREB2	comp38103_c1	not DE	n.a.	n.a.	n.a.	comp32151_c0_seq1	↓ 4h	2,27	-3,67	1,24E-05
							↓ 24h	2,27	-6,19	8,08E-14
dCREBA	comp37729_c7	not DE	n.a.	n.a.	n.a.	comp21911_c1_seq1	↑ 0h	5,45	0,77	2,46E-04
dDA1	comp37078_c0_seq2	↓ 4h	2,89	-1,38	1,79E-03	comp32875_c0_seq2	↓ 4h	2,44	-4,38	8,59E-12
Dunce	comp32729_c1_seq5	↑ 4h	5,71	0,64	3,83E-05	comp34739_c4_seq3	↓ 4h	5,36	-0,76	4,53E-04
eIF-5C	comp40716_c0_seq1	↑ 4h	7,66	0,40	3,71E-04	comp36025_c0_seq1	↑ 24h	7,24	0,57	4,37E-04
Fasciclin 1	comp39514_c1	not DE	n.a.	n.a.	n.a.	comp34589_c0_seq1	↓ 24h	5,68	-0,67	7,61E-04
Fasciclin 2	comp34025_c1_seq3	↑ 0h	5,59	0,63	1,03E-04	comp32884_c1	not DE	n.a.	n.a.	n.a.
		↑ 4h	5,59	0,68	2,39E-05					
		↑ 24h	5,59	0,62	1,15E-04					
Fasciclin 3	comp37859_c0_seq14	↓ 0h	1,54	-10,95	1,39E-15	comp32890_c0_seq8 (1)	↑ 4h	1,86	3,19	2,52E-07
							↑ 24h	1,86	2,53	2,91E-05
						comp32890_c0_seq10 (2)	↑ 4h	1,90	2,66	5,61E-06
							↑ 24h	1,90	2,14	2,66E-04
						comp32890_c0_seq12 (3)	↓ 24h	4,00	-1,04	1,76E-03
						comp32890_c0_seq13 (4)	↑ 4h	-0,27	3,66	3,21E-04
						comp32890_c0_seq15 (5)	↓ 4h	2,82	-1,63	5,11E-04
FMRP	comp34913_c0_seq6	↑ 0h	5,12	0,67	6,65E-04	comp30902_c0	not DE	n.a.	n.a.	n.a.
Foraging	comp27264_c0	not DE	n.a.	n.a.	n.a.	comp22710_c0_seq2	↓ 24h	4,03	-1,05	1,72E-03
G-sα60A	comp35901_c1_seq2	↑ 24h	0,05	3,62	2,05E-04	comp18726_c0	not DE	n.a.	n.a.	n.a.
Hopscotch	comp25200_c0_seq2	↓ 0h	2,30	-2,92	7,40E-07	comp22884_c0	not DE	n.a.	n.a.	n.a.
Linotte	comp33720_c1_seq2	↓ 0h	1,24	-5,95	2,78E-07	comp19883_c0	not DE	n.a.	n.a.	n.a.
NF1	comp32412_c0	not DE	n.a.	n.a.	n.a.	comp33011_c0_seq3 (1)	↑ 24h	3,83	1,78	7,03E-06
						comp33011_c0_seq5 (2)	↑ 24h	3,03	2,15	2,41E-06

						comp33011_c0_seq6 (3)	↑ 24h	5,08	1,99	7,32E-15
Nmda1	comp13931_c0	not DE	n.a.	n.a.	n.a.	comp21393_c0_seq1	↑ 24h	4,74	0,82	2,25E-03
NMDAR1	comp39881_c3_seq1	↑ 0h	-0,44	4,20	5,82E-04	comp22369_c0	not DE	n.a.	n.a.	n.a.
NMDAR2	comp39633_c6	not DE	n.a.	n.a.	n.a.	comp30975_c0_seq3	↓ 4h	0,74	-2,31	9,22E-04
Orb2	comp38340_c1_seq3	↑ 4h	2,95	2,34	2,27E-07	comp32650_c0	not DE	n.a.	n.a.	n.a.
Pale	comp14977_c0_seq1	↑ 4h	7,32	0,51	1,92E-05	comp29518_c0_seq1	↓ 24h	5,56	-1,74	3,56E-16
		↓ 24h	7,32	-1,49	1,88E-33					
PKA3	comp15756_c0_seq1	↑ 4h	3,74	1,12	3,90E-04	comp39532_c0_seq1	↑ 4h	5,86	1,02	8,81E-08
PKA-R2	comp36737_c1_seq3 (1)	↑ 24h	5,79	0,50	1,09E-03	comp34612_c3_seq35	↓ 4h	5,86	-0,67	4,52E-04
	comp36737_c1_seq4 (2)	↑ 4h	3,50	1,41	6,27E-05					
	comp36737_c1_seq9 (3)	↑ 4h	6,42	0,75	3,10E-08					
		↑ 24h	6,42	0,56	4,04E-05					
	comp36737_c1_seq10 (4)	↓ 4h	6,22	-0,57	4,61E-05					
	comp36737_c1_seq11 (5)	↑ 4h	6,68	0,44	6,30E-04					
PKC δ	comp36706_c0_seq9 (1)	↓ 4h	0,47	-2,41	1,78E-03	comp33557_c0	not DE	n.a.	n.a.	n.a.
	comp36706_c0_seq12 (2)	↑ 4h	0,99	2,11	9,45E-04					
Pumilio	comp38467_c2_seq2	↓ 4h	6,06	-0,60	3,05E-05	comp28801_c0	not DE	n.a.	n.a.	n.a.
Src64B	comp35269_c1_seq1	↓ 0h	0,16	-3,39	8,61E-04	comp32598_c1	not DE	n.a.	n.a.	n.a.
Staufen	comp32934_c0_seq2	↓ 0h	4,19	-1,51	3,38E-07	comp29323_c0	not DE	n.a.	n.a.	n.a.
Su(H)	comp35504_c1_seq1 (1)	↑ 0h	4,08	1,01	7,87E-04	comp25570_c0	not DE	n.a.	n.a.	n.a.
		↑ 4h	4,08	1,07	3,82E-04	comp21669_c1	not DE	n.a.	n.a.	n.a.
	comp35504_c1_seq2 (2)	↑ 0h	-0,41	4,50	1,80E-04					
	comp25453_c1	not DE	n.a.	n.a.	n.a.					
Tdc1	comp38213_c2	not DE	n.a.	n.a.	n.a.	comp35179_c0_seq7	↓ 24h	0,23	-3,72	8,61E-05
						comp35148_c0	not DE	n.a.	n.a.	n.a.
Tequila	comp36628_c1_seq1	↓ 4h	4,54	-1,60	9,32E-10	comp31122_c2	not DE	n.a.	n.a.	n.a.

References

Akalal DBG, Yu DH, Davis RL (2011) The long-term memory trace formed in the *Drosophila* α/β mushroom body neurons is abolished in long-term memory mutants. *Journal of Neuroscience* **31**, 5643-5647.

Alberini CM (2009) Transcription factors in long-term memory and synaptic plasticity. *Physiological Reviews* **89**, 121-145.

Banerjee P, Schoenfeld BP, Bell AJ, *et al*. (2010) Short- and long-term memory are modulated by multiple isoforms of the fragile X mental retardation protein. *Journal of Neuroscience* **30**, 6782-6792.

Bevilaqua LR, Medina JH, Izquierdo I, Cammarota M (2005) Memory consolidation induces n-methyl-d-aspartic acid-receptor- and Ca2+/calmodulin-dependent protein kinase II-dependent modifications in alpha-amino-3-hydroxy-5-methylisoxazole-4-propionic acid receptor properties. *Neuroscience* **136**, 397-403.

Burke CJ, Huetteroth W, Owald D, *et al*. (2012) Layered reward signalling through octopamine and dopamine in *Drosophila*. *Nature* **492**, 433-437.

Copf T, Goguel V, Lampin-Saint-Amaux A, Scaplehorn N, Preat T (2011) Cytokine signaling through the JAK/STAT pathway is required for long-term memory in *Drosophila*. *Proceedings of the National Academy of Sciences of the United States of America* **108**, 8059-8064.

da Cruz AB, Wentzell J, Kretzschmar D (2008) Swiss cheese, a protein involved in progressive neurodegeneration, acts as a noncanonical regulatory subunit for PKA-C3. *Journal of Neuroscience* **28**, 10885-10892.

Dubnau J, Chiang AS, Grady L, *et al*. (2003) The staufen/pumilio pathway is involved in *Drosophila* long-term memory. *Current Biology* **13**, 286-296.

Eickhoff R, Bicker G (2012) Developmental expression of cell recognition molecules in the mushroom body and antennal lobe of the locust *Locusta migratoria*. *Journal of comparative neurology* **520**, 2021-2040.

Gallagher HC, Murphy KJ, Foley AG, Regan CM (2001) Protein kinase C δ regulates neural cell adhesion molecule polysialylation state in the rat brain. *Journal of Neurochemistry* **77**, 425-434.

Griffith LC, Wang J, Zhong Y, Wu CF, Greenspan RJ (1994) Calcium/calmodulin-dependent protein kinase II and potassium channel subunit EAG similarly affect plasticity in *Drosophila*. *Proceedings of the National Academy of Sciences of the United States of America* **91**, 10044-10048.

Hearn MG, Ren Y, McBride EW, *et al*. (2002) A *Drosophila* dopamine 2-like receptor: Molecular characterization and identification of multiple alternatively spliced variants. *Proceedings of the National Academy of Sciences of the United States of America* **99**, 14554-14559.

Hirano Y, Masuda T, Naganos S, *et al*. (2013) Fasting launches CRTC to facilitate long-term memory formation in *Drosophila*. *Science* **339**, 443-446.

Homberg U, Seyfarth J, Binkle U, Monastirioti M, Alkema MJ (2013) Identification of distinct tyraminergic and octopaminergic neurons innervating the central complex of the desert locust, *Schistocerca gregaria*. *Journal of comparative neurology* **521**, 2025-2041.

Ishimoto H, Wang Z, Rao Y, Wu CF, Kitamoto T (2013) A novel role for ecdysone in *Drosophila* conditioned behavior: linking GPCR-mediated non-canonical steroid action to cAMP signaling in the adult brain. *PLoS Genetics* **9**, e1003843.

Iyer SC, Iyer EPR, Meduri R, *et al*. (2013) Cut, via CrebA, transcriptionally regulates the COPII secretory pathway to direct dendrite development in *Drosophila*. *Journal of Cell Science* **126**, 4732-4745.

Johnson O, Becnel J, Nichols CD (2011) Serotonin receptor activity is necessary for olfactory learning and memory in *Drosophila melanogaster*. *Neuroscience* **192**, 372-381.

Keene AC, Waddell S (2007) *Drosophila* olfactory memory: single genes to complex neural circuits. *Nature Reviews Neuroscience* **8**, 341-354.

Knapek S, Sigrist S, Tanimoto H (2011) Bruchpilot, a synaptic active zone protein for anesthesia-resistant memory. *Journal of Neuroscience* **31**, 3453-3458.

Liu WW, Wilson RI (2013) Glutamate is an inhibitory neurotransmitter in the *Drosophila* olfactory system. *Proceedings of the National Academy of Sciences of the United States of America* **110**, 10294-10299.

Malik BR, Gillespie JM, Hodge JJL (2013) CASK and CaMKII function in the mushroom body α'/β' neurons during *Drosophila* memory formation. *Frontiers in Neural Circuits* **7**, 52.

Meiser J, Weindl D, Hiller K (2013) Complexity of dopamine metabolism. *Cell Communication and Signaling* **11**, 34.

Mery F, Belay AT, So AKC, Sokolowski MB, Kawecki TJ (2007) Natural polymorphism affecting learning and memory in *Drosophila*. *Proceedings of the National Academy of Sciences of the United States of America* **104**, 13051-13055.

Nagaya Y, Kutsukake M, Chigusa SI, Komatsu A (2002) A trace amine, tyramine, functions as a neuromodulator in *Drosophila melanogaster*. *Neuroscience Letters* **329**, 324-328.

Pagani MR, Oishi K, Gelb BD, Zhong Y (2009) The phosphatase SHP2 regulates the spacing effect for long-term memory induction. *Cell* **139**, 186-198.

Pai TP, Chen CC, Lin HH, *et al*. (2013) *Drosophila* ORB protein in two mushroom body output neurons is necessary for long-term memory formation. *Proceedings of the National Academy of Sciences of the United States of America* **110**, 7898-7903.

Riedel G, Platt B, Micheau J (2003) Glutamate receptor function in learning and memory. *Behavioural Brain Research* **140**, 1-47.

Schmitt JM, Guire ES, Saneyoshi T, Soderling TR (2005) Calmodulin-dependent kinase kinase/calmodulin kinase I activity gates extracellular-regulated kinase-dependent long-term potentiation. *Journal of Neuroscience* **25**, 1281-1290.

Schwaerzel M, Jaeckel A, Mueller U (2007) Signaling at A-kinase anchoring proteins organizes anesthesia-sensitive memory in *Drosophila*. *Journal of Neuroscience* **27**, 1229-1233.

Shuai YC, Lu BY, Hu Y, *et al*. (2010) Forgetting is regulated through Rac activity in *Drosophila*. *Cell* **140**, 579-589.

Song QX, Sun K, Shuai Y, *et al*. (2009) Suppressor of Hairless is required for long-term memory formation in *Drosophila*. *Journal of Neurogenetics* **23**, 405-411.

Tejedor F, Zhu XR, Kaltenbach E, *et al*. (1995) Minibrain: a new protein kinase family involved in postembryonic neurogenesis in *Drosophila*. *Neuron* **14**, 287-301.

Thiels E, Kanterewicz BI, Knapp LT, Barrionuevo G, Klann E (2000) Protein phosphatase-mediated regulation of protein kinase C during long-term depression in the adult hippocampus in vivo. *Journal of Neuroscience* **20**, 7199-7207.

Xia S, Miyashita T, Fu T-F, *et al*. (2005) NMDA receptors mediate olfactory learning and memory in *Drosophila*. *Current Biology* **15**, 603-615.

Zisopoulou S, Asimaki O, Leondaritis G, et al. (2013) PKC-ε activation is required for recognition memory in the rat. *Behavioural Brain Research* **253**, 280-289.

--Table S7--

Differentially expressed genes with an opposed expression pattern after conditioning in *N. vitripennis* and *N. giraulti*. (a) Genes of which all transcript variants have an opposed expression pattern between the two *Nasonia* species are given (↑ = upregulated, ↓ = downregulated). For each DE transcript, the log2-count per million (cpm), log2-fold change (FC), and the level of significance (*P*) are given for the time points at which the transcripts are differentially expressed. (b) GO enrichment analyses of the genes with an opposed expression pattern was done using Blast2go (Fisher's exact test, P < 0.05). The two categories of antisense transcripts were analysed separately: 'antisense2protein' transcripts that have a hit to a *N. vitripennis* protein and 'antisense2sense' transcripts that have a hit to a sense transcript only. Generic GOSlim categories and the Blast2go option 'reduce to most specific terms' were used to limit the number of GO term categories. The enriched GO terms (GO ID and Term), the category of the GO term (C = cellular component, F = molecular function, P = biological process), their *P*-value and the number of genes in the test-set and the reference-set are given.

a. Genes with an opposing expression pattern between *N. vitripennis* and *N. giraulti*

		N. vitripennis					*N. giraulti*				
**OGS 2.0 ID**	**Gene description**	**Transcript ID**	**Time-point**	**Log2-CPM**	**Log2-FC**	***P***	**Transcript ID**	**Time-point**	**Log2-CPM**	**Log2-FC**	***P***
Nasvi2EG000127	Proton-coupled folate transporter	comp13904_c0_seq1	↑0h	6,7069	0,5311	5,09E-05	comp28717_c0_seq2	↓24h	2,4579	-2,0411	3,50E-04
			↑4h	6,7069	0,5510	2,42E-05					
			↑24h	6,7069	0,4297	1,01E-03					
Nasvi2EG000216	Purine nucleoside phosphorylase	comp40525_c0_seq1	↑4h	8,7895	0,3614	1,79E-04	comp35798_c0_seq1	↓4h	7,9386	-0,7106	8,97E-06
								↓24h	7,9386	-0,6574	4,46E-05
Nasvi2EG000732	Cytochrome P450 4C1	comp40995_c0_seq1	↑24h	4,6076	0,7762	1,75E-03	comp38976_c0_seq1	↓4h	6,7087	-0,7252	1,79E-05
Nasvi2EG000852	Aquaporin AQPAn.G	comp36203_c2_seq1	↑4h	7,5213	0,4635	6,41E-05	comp31207_c1_seq2	↓24h	7,5715	-0,5738	3,44E-04
							comp31207_c1_seq3	↓24h	8,3098	-0,6180	1,32E-04

							comp31207_c1_seq6	↓24h	2,9405	-1,9054	4,42E-05
Nasvi2EG001309	Unknown	comp37153_c0_seq6	↑24h	6,0878	0,4611	1,20E-03	comp30474_c0_seq1	↓4h	6,1580	-0,6405	4,02E-04
		comp37153_c0_seq2	↑24h	7,0832	0,4216	6,38E-04		↓24h	6,1580	-0,7439	4,35E-05
							comp30474_c0_seq5	↓4h	3,8013	-2,9271	2,33E-14
Nasvi2EG001615	Spectrin, beta chain	comp39193_c2_seq10	↑0h	3,6436	1,4334	2,56E-05	comp32657_c0_seq10	↓4h	5,9936	-0,6304	6,86E-04
Nasvi2EG003098	Maleylacetoacetate isomerase 2	comp37898_c3_seq5	↑0h	1,7246	3,1754	1,03E-05	comp37698_c0_seq1	↓4h	6,6049	-0,7011	4,05E-05
			↑24h	1,7246	4,2428	1,96E-07		↓24h	6,6049	-0,5551	1,16E-03
Nasvi2EG003481	Muscle-specific protein	comp39798_c0_seq34	↑0h	3,8536	1,0416	8,85E-04	comp34922_c0_seq71	↓24h	3,1213	-1,9486	8,17E-05
		comp39798_c0_seq14	↑24h	4,0107	0,9969	8,80E-04					
		comp39798_c0_seq32	↑24h	4,0594	0,9612	1,13E-03					
Nasvi2EG003488	Xanthine dehydrogenase	comp41097_c0_seq1	↑4h	7,7478	0,4003	3,61E-04	comp18900_c0_seq1	↓4h	7,5104	-0,6998	1,24E-05
								↓24h	7,5104	-0,6908	1,67E-05
Nasvi2EG004756	snRNA-activating protein complex, subunit 1	comp38773_c0_seq14	↑4h	1,0423	3,0467	2,81E-05	comp34285_c0_seq1	↓4h	0,9164	-0,0080	9,89E-01
Nasvi2EG006622	Fem-1 protein A	comp31321_c0_seq3	↑0h	3,4337	1,2399	3,01E-04	comp33585_c2_seq5	↓0h	4,7568	-1,1566	2,01E-05
Nasvi2EG006723	Sulfotransferase family cytosolic 1B member	comp39757_c16_seq16	↑0h	0,3092	4,4642	1,37E-05	comp34937_c1_seq61	↓24h	-0,5635	-4,6934	1,73E-04
			↑4h	0,3092	2,8306	1,42E-03					
Nasvi2EG006827	Endothelin-converting enzyme 1	comp39911_c0_seq2	↑4h	7,0332	0,4094	9,74E-04	comp34987_c1_seq13	↓0h	4,9543	-0,8763	4,98E-04
Nasvi2EG007296	Furry	comp35259_c0_seq2	↑4h	5,7147	0,5322	5,11E-04	comp31740_c0_seq10	↓24h	1,3000	-2,6760	3,28E-05
							comp31740_c0_seq13	↓24h	2,5558	-2,4910	1,54E-05
							comp31740_c0_seq16	↓24h	1,6094	-2,0402	9,37E-04
Nasvi2EG007947	Guanine nucleotide-binding protein G, subunit alpha	comp39607_c4_seq2	↑0h	6,0399	0,5101	3,73E-04	comp34920_c5_seq3	↓24h	1,4566	-2,5243	2,73E-05
Nasvi2EG008711	Bifunctional purine biosynthesis protein PURH	comp40780_c0_seq1	↑4h	7,1800	1,5877	1,94E-37	comp36134_c0_seq1	↓0h	6,9850	-0,5949	3,11E-04
Nasvi2EG008828	Unknown	comp38853_c1_seq6	↑0h	1,4130	2,3081	1,87E-04	comp34206_c1_seq1	↓24h	0,4649	-9,6315	5,56E-09
			↑4h	1,4130	1,9996	1,00E-03	comp34206_c1_seq11	↓24h	-0,4594	-8,4590	1,87E-05
							comp34206_c1_seq2	↓24h	-0,0084	-8,8410	1,92E-06
							comp34206_c1_seq4	↓24h	-0,2745	-9,0885	1,49E-06
Nasvi2EG009166	Acyl-CoA Delta desaturase	comp26940_c0_seq1	↑0h	8,8851	0,5290	2,95E-08	comp21842_c1_seq1	↓24h	10,1861	-0,7186	7,67E-05
Nasvi2EG009221	Peroxidase	comp37834_c0_seq8	↑24h	6,1144	0,4788	6,90E-04	comp33499_c0_seq5	↓0h	7,4232	-0,9197	1,33E-08

		comp37834_c0_seq1	↑4h	5,2015	0,7017	2,04E-04	comp33499_c0_seq14	↓0h	5,3673	-0,8465	9,96E-05
			↑24h	5,2015	0,9112	1,51E-06					
Nasvi2EG010628	Alanine-glyoxylate aminotransferase 2	comp14128_c0_seq1	↑0h	6,6830	0,4779	2,64E-04	comp28352_c1_seq1	↓24h	5,4888	-0,7375	4,11E-04
		comp14128_c0_seq2	↑0h	6,9205	0,7802	8,51E-10					
			↑24h	6,9205	0,3921	1,97E-03					
Nasvi2EG010669	Long-chain-fatty-acid--CoA ligase ACSBG2	comp14643_c0_seq2	↑24h	7,6893	0,3913	5,42E-04	comp22964_c0_seq1	↓4h	8,2352	-0,8118	4,80E-07
							comp22964_c0_seq2	↓4h	6,4269	-0,6457	2,13E-04
Nasvi2EG010717	5-formyltetrahydrofolate cyclo-ligase	comp41384_c0_seq1	↑24h	4,7909	0,9295	4,58E-05	comp36886_c0_seq1	↓0h	5,1094	-0,8410	3,60E-04
Nasvi2EG011011	C-1-tetrahydrofolate synthase	comp37840_c0_seq1	↑4h	2,7699	1,7655	9,31E-05	comp31204_c0_seq1	↓4h	4,8041	-1,1443	1,48E-05
		comp37840_c0_seq2	↑4h	6,7378	0,6690	2,61E-07					
			↑24h	6,7378	0,6945	8,98E-08					
Nasvi2EG011498	cAMP-specific 3',5'-cyclic phosphodiesterase	comp32729_c1_seq5	↑4h	5,7126	0,6366	3,83E-05	comp34739_c4_seq3	↓4h	5,3623	-0,7594	4,53E-04
		comp34929_c0_seq3	↑4h	0,5474	3,4909	1,53E-05					
Nasvi2EG011895	Mediator of cell motility 1	comp29544_c0_seq1	↑0h	2,0136	2,2217	3,24E-04	comp31811_c0_seq4	↓0h	3,3005	-2,1227	6,49E-07
							comp31811_c0_seq5	↓0h	0,8986	-2,8738	3,68E-05
Nasvi2EG012254	Cytoplasmic dynein 1 intermediate chain	comp38462_c2_seq7	↑0h	-0,8002	7,8512	1,60E-04	comp34439_c0_seq11	↓24h	2,6894	-2,5614	5,46E-07
			↑4h	-0,8002	7,9714	1,80E-04	comp34439_c0_seq2	↓24h	2,3932	-1,9182	4,40E-04
			↑24h	-0,8002	7,9852	9,87E-05	comp34439_c0_seq22	↓24h	1,4300	-2,2943	1,60E-04
Nasvi2EG012394	Unknown	comp38936_c0_seq3	↑24h	0,2613	2,8201	8,68E-04	comp34737_c1_seq2	↓4h	0,6451	-2,4073	1,09E-03
		comp38936_c2_seq11	↑24h	0,4979	3,3490	7,20E-05	comp34737_c1_seq41	↓4h	0,7977	-2,3770	6,99E-04
		comp38936_c2_seq20	↑24h	0,9498	2,6011	1,41E-04	comp34737_c1_seq47	↓4h	1,4807	-2,5521	4,28E-05
							comp34737_c1_seq44	↓24h	3,1803	-1,4567	4,74E-04
							comp34737_c1_seq58	↓24h	1,4696	-2,1898	2,79E-04
Nasvi2EG014157	Anterior Fat body protein	comp39564_c2_seq4	↑0h	0,6705	2,8523	1,95E-04	comp29801_c0_seq2	↓24h	6,8887	-0,5665	6,65E-04
			↑24h	0,6705	3,2455	1,47E-04					
Nasvi2EG015987	Anion exchange protein 2	comp37382_c1_seq10	↑4h	4,9209	0,6696	1,87E-03	comp30734_c0_seq3	↓24h	3,9942	-1,2383	2,72E-04
							comp30734_c0_seq7	↓24h	4,9986	-1,1159	8,16E-06
Nasvi2EG017772	Metaxin-1	comp36033_c0_seq1	↑0h	0,0692	3,7700	3,12E-04	comp31841_c0_seq7	↓24h	0,6855	-2,8691	2,58E-04
Nasvi2EG019460	Upstream-binding protein 1	comp38559_c3_seq6	↑0h	4,9023	1,0739	8,59E-07	comp33597_c4_seq1	↓4h	3,7430	-1,1385	1,52E-03

							comp33597_c4_seq10	↓4h	5,4716	-0,6714	1,31E-03
								↓24h	5,4716	-0,7553	3,74E-04
							comp33597_c4_seq13	↓4h	5,8013	-0,7799	5,57E-05
Nasvi2EG019465	Isochorismatase domain-containing protein 1	comp40396_c0_seq1	↑4h	7,8254	0,9853	1,25E-18	comp35904_c0_seq1	↓24h	8,3613	-0,6282	1,07E-04
Nasvi2EG020039	Unknown	comp40067_c0_seq8	↑0h	2,2063	3,5026	2,73E-08	comp35430_c0_seq24	↓24h	1,3379	-2,7952	1,30E-05
Nasvi2EG020115	Ribose-phosphate pyrophosphokinase 1	comp14056_c0_seq2	↑4h	8,2703	0,5364	2,72E-07	comp17986_c0_seq2	↓4h	7,8659	-0,6407	5,96E-05
							comp17986_c0_seq1	↓4h	6,6072	-0,5409	1,52E-03
Nasvi2EG023114	Phosphatidylinositol 3-kinase, catalytic subunit type 3	comp39910_c1_seq23	↑0h	-0,1315	3,8006	3,27E-04	comp32330_c0_seq5	↓0h	-0,1374	-4,6426	1,51E-04
Nasvi2EG023755	Dipeptidyl aminopeptidase protein 6	comp37608_c1_seq5	↑0h	2,6223	1,9552	8,54E-05	comp33935_c0_seq2	↓24h	4,0781	-1,2197	3,31E-04
		comp37608_c1_seq9	↑0h	0,4486	3,4380	1,38E-04	comp33935_c0_seq3	↓24h	3,0136	-2,3908	3,05E-06
			↑4h	0,4486	3,8889	1,27E-05					
			↑24h	0,4486	3,5492	4,87E-05					
Nasvi2EG026831	Spire	comp38647_c0_seq23	↑4h	3,0374	1,6528	4,45E-05	comp34537_c1_seq8	↓24h	1,2631	-1,9727	1,11E-03
Nasvi2EG027277	Cytochrome P450 6a2	comp39892_c0_seq10	↑0h	8,6131	0,5910	3,41E-09	comp32522_c0_seq6	↓0h	-0,5031	-4,5833	2,66E-04
			↑4h	8,6131	0,8754	2,29E-18		↓24h	-0,5031	-8,5972	9,80E-06
			↑24h	8,6131	0,8008	1,33E-15					
Nasvi2EG036430	Muscle-specific protein	comp40134_c1_seq1	↑24h	10,2374	0,3063	5,97E-05	comp35565_c0_seq1	↓24h	10,2398	-0,6458	3,84E-04
Nasvi2EG037293	PH domain leucine-rich repeat-containing protein phosphatase 2	comp28608_c0_seq1	↑24h	1,8457	1,8606	1,33E-03	comp32316_c0_seq3	↓4h	1,3113	-2,6563	2,54E-05
Nasvi2EG001269	Esterase FE4	comp26462_c0_seq1	↓0h	2,9869	-1,4849	7,20E-04	comp31542_c0_seq3	↑24h	4,3534	1,5680	4,37E-05
							comp31701_c1_seq2	↑24h	4,2659	2,4546	6,11E-11
Nasvi2EG001270	Esterase FE4	comp14324_c0_seq1	↓24h	2,0213	-2,4780	2,01E-04	comp31701_c1_seq1	↑24h	3,7281	2,7139	6,80E-09
Nasvi2EG002254	Guanylate cyclase	comp39271_c0_seq35	↓0h	0,7959	-2,5988	2,14E-04	comp33578_c0_seq11	↑4h	2,5787	1,9587	3,11E-04
							comp33578_c0_seq4	↑4h	1,0254	2,3493	1,95E-04
Nasvi2EG002407	spermine synthase	comp32637_c0_seq3	↓24h	0,4953	-3,7293	3,13E-05	comp34397_c0_seq15	↑24h	1,9269	1,9793	2,43E-03
		comp32637_c0_seq8	↓24h	1,5050	-2,3045	2,02E-04					
Nasvi2EG002573	ELAV protein 2	comp38945_c3_seq17	↓24h	0,2727	-4,5553	4,31E-05	comp34728_c5_seq17	↑0h	-0,0602	7,5419	3,57E-04
								↑4h	-0,0602	9,4544	1,50E-06
								↑24h	-0,0602	10,9108	1,01E-08

Nasvi2EG003024	dnaJ protein 1	comp32266_c1_seq1	↓0h	6,2099	-0,4914	5,08E-04	comp36805_c0_seq1	↑24h	6,4502	0,7793	7,86E-06
Nasvi2EG003499	Glycosylphosphatidylinositol anchor attachment 1 protein	comp29211_c0_seq2	↓24h	3,9431	-1,1763	2,49E-04	comp30546_c1_seq2	↑4h	3,3922	1,3866	7,43E-04
								↑24h	3,3922	1,3136	9,26E-04
Nasvi2EG003599	Unknown	comp37859_c0_seq14	↓0h	1,5412	-10,951	1,39E-15	comp32890_c0_seq8	↑4h	1,8552	3,1942	2,52E-07
								↑24h	1,8552	2,5314	2,91E-05
							comp32890_c0_seq10	↑4h	1,8972	2,6585	5,61E-06
								↑24h	1,8972	2,1361	2,66E-04
							comp32890_c0_seq13	↑4h	-0,2713	3,6577	3,21E-04
Nasvi2EG003744	Unknown	comp40897_c0_seq1	↓24h	5,3959	-0,6250	2,75E-04	comp37571_c0_seq1	↑24h	6,8119	0,6679	6,49E-05
Nasvi2EG003940	Cytochrome B5-related protein	comp14037_c0_seq1	↓0h	4,7125	-0,9974	7,16E-05	comp33057_c0_seq3	↑24h	7,1907	0,7439	5,41E-06
			↓4h	4,7125	-1,6963	1,45E-11					
Nasvi2EG003990	Nasonin-2	comp40154_c1_seq1	↓0h	7,6222	-0,7047	1,13E-09	comp35720_c0_seq1	↑24h	9,1613	1,7870	4,97E-23
			↓24h	7,6222	-0,4770	4,00E-05					
Nasvi2EG003991	Nasonin-1	comp40297_c0_seq1	↓24h	7,5581	-0,4455	1,20E-04	comp35728_c0_seq1	↑24h	7,5913	1,6185	1,96E-22
Nasvi2EG004153	Unknown	comp32393_c0_seq1	↓4h	5,7582	-0,5713	1,62E-04	comp29021_c0_seq1	↑24h	5,3187	0,9204	7,70E-05
			↓24h	5,7582	-0,5804	1,32E-04					
Nasvi2EG004317	Unknown	comp35614_c0_seq4	↓0h	0,5143	-2,7550	4,32E-04	comp21614_c0_seq1	↑0h	0,2144	8,9037	1,75E-07
			↓4h	0,5143	-3,0620	2,63E-04		↑4h	0,2144	8,6565	4,28E-07
			↓24h	0,5143	-2,9188	1,49E-04		↑24h	0,2144	10,0737	4,02E-10
Nasvi2EG004554	Rac GTPase-activating protein	comp39295_c1_seq3	↓0h	8,6588	-0,4800	1,25E-06	comp32552_c2_seq1	↑24h	9,5674	0,7218	3,72E-05
Nasvi2EG005473	Nahymenoptaecin-1	comp39002_c0_seq1	↓0h	6,3673	-0,6754	1,07E-06	comp34500_c1_seq2	↑24h	8,7915	1,1675	8,29E-12
		comp39002_c0_seq2	↓0h	7,0258	-0,6703	9,21E-08	comp34500_c1_seq1	↑24h	7,6435	1,1301	1,32E-11
Nasvi2EG007441	1-phosphatidylinositol-4.5-bisphosphate phosphodiesterase	comp38606_c0_seq20	↓0h	4,5595	-1,1185	1,64E-05	comp33641_c1_seq15	↑24h	2,4838	1,6322	1,76E-03
		comp38606_c0_seq1	↓0h	5,0414	-0,7995	1,18E-04					
Nasvi2EG007789	Lipid storage droplets surface-binding protein 2	comp35521_c0_seq1	↓4h	5,4424	-0,7769	3,97E-06	comp27767_c0_seq1	↑24h	5,3537	0,7482	6,43E-04
		comp35521_c0_seq3	↓4h	4,6868	-1,2246	5,64E-07					
Nasvi2EG007954	Uridine phosphorylase	comp26800_c0_seq1	↓24h	6,0478	-0,4716	1,08E-03	comp31942_c0_seq6	↑4h	6,4696	0,7788	8,14E-06
Nasvi2EG008141	lethal(2)essential for life protein	comp42085_c0_seq1	↓4h	3,7406	-1,0435	9,63E-04	comp38460_c0_seq1	↑24h	4,7223	1,3300	1,75E-05
			↓24h	3,7406	-0,9878	1,78E-03					

Nasvi2EG008392	Antimicrobial peptide Def1-1	comp40158_c0_seq1	↓0h	5,9741	-0,5585	1,45E-04	comp36123_c0_seq1	↑4h	9,2655	0,3621	3,80E-02
			↓24h	5,9741	-0,8587	1,85E-08		↑24h	9,2655	1,7060	6,54E-22
Nasvi2EG008393	Antimicrobial peptide Def1-1	comp40145_c0_seq1	↓0h	7,5744	-0,6047	2,01E-07	comp35717_c0_seq1	↑4h	8,7908	0,7092	2,39E-05
								↑24h	8,7908	1,5526	1,39E-19
Nasvi2EG008872	HEAT repeat-containing protein C7orf27	comp26504_c0_seq1	↓0h	3,5335	-1,5680	5,80E-06	comp24079_c0_seq5	↑4h	3,5910	1,4159	2,59E-04
								↑24h	3,5910	2,4569	2,36E-09
Nasvi2EG009157	Unknown	comp16168_c0_seq2	↓0h	6,1225	-0,5021	5,60E-04	comp35771_c0_seq1	↑24h	7,0058	0,5048	2,18E-03
			↓4h	6,1225	-0,5678	1,29E-04					
Nasvi2EG009349	Nucleoporin p58/p45	comp35160_c0_seq3	↓0h	-0,0990	-8,9405	1,78E-06	comp29535_c0_seq6	↑4h	-0,2613	10,5048	1,23E-09
Nasvi2EG009908	Low-density lipoprotein receptor-related protein	comp41424_c0_seq1	↓4h	6,3567	-0,5652	3,42E-05	comp27113_c0_seq2	↑24h	3,0478	1,4329	1,16E-03
Nasvi2EG011261	Enolase-phosphatase E1	comp26470_c0_seq1	↓4h	5,0690	-0,7005	4,18E-04	comp31694_c0_seq1	↑24h	5,8149	1,4369	3,12E-11
			↓24h	5,0690	-0,7317	2,41E-04					
Nasvi2EG011349	Hydroxysteroid dehydrogenase protein 1	comp39591_c0_seq17	↓4h	6,0675	-0,4759	8,54E-04	comp30098_c0_seq1	↑24h	5,4626	1,0831	2,60E-07
		comp39591_c0_seq25	↓4h	7,7508	-0,6722	2,43E-09	comp30098_c0_seq3	↑24h	9,1196	0,6349	1,74E-04
			↓24h	7,7508	-0,4110	2,58E-04	comp30098_c0_seq4	↑24h	5,0949	1,0113	1,92E-05
		comp39591_c0_seq29	↓4h	4,6606	-1,1415	3,38E-06					
		comp39591_c0_seq36	↓4h	3,9106	-1,3143	2,67E-05					
		comp39591_c0_seq39	↓4h	4,5981	-0,8559	7,39E-04					
		comp39591_c0_seq57	↓4h	3,8491	-1,1267	3,21E-04					
		comp39591_c0_seq67	↓4h	3,8336	-1,0350	1,08E-03					
Nasvi2EG011454	Unknown	comp40253_c0_seq1	↓24h	8,3468	-0,7487	6,40E-13	comp35622_c0_seq1	↑24h	7,9758	0,7714	1,49E-06
Nasvi2EG011714	Unknown	comp27053_c0_seq1	↓0h	7,2578	-0,7950	3,63E-10	comp36024_c0_seq1	↑24h	7,1806	0,5804	9,85E-04
		comp27053_c0_seq2	↓0h	4,0872	-1,9238	3,65E-09					
Nasvi2EG011809	RUN and FYVE domain-containing protein 3	comp36818_c0_seq4	↓4h	1,7007	-2,3361	1,74E-04	comp32758_c0_seq5	↑0h	1,2212	2,4938	3,09E-04
							comp32758_c0_seq8	↑0h	1,2747	2,5410	1,87E-04
Nasvi2EG012170	Paired amphipathic helix protein Sin3a	comp36674_c0_seq4	↓24h	3,7459	-0,9991	1,86E-03	comp33148_c1_seq4	↑24h	3,1828	1,5101	2,78E-04
Nasvi2EG012199	Niemann-Pick C1 protein	comp35712_c1_seq8	↓4h	3,7408	-1,0419	1,38E-03	comp30364_c1_seq3	↑24h	3,8478	1,1794	1,05E-03
		comp35712_c1_seq1	↓4h	3,3485	-1,1412	1,47E-03					
Nasvi2EG012478	Aminopeptidase N	comp33902_c0_seq4	↓24h	5,0147	-0,7403	8,97E-04	comp14087_c0_seq1	↑24h	3,8536	1,5217	1,53E-05

							comp14087_c0_seq2	↑24h	6,4501	0,6544	1,96E-04
Nasvi2EG012746	Counting factor associated proteinD	comp35299_c3_seq2	↓4h	9,1004	-0,3548	1,18E-04	comp21988_c0_seq2	↑24h	6,6130	1,1764	3,03E-11
Nasvi2EG013276	Nahymenoptaecin-2	comp26972_c0_seq1	↓24h	4,3834	-2,1763	6,07E-12	comp33091_c1_seq1	↑4h	7,6152	1,6694	1,42E-22
		comp26972_c0_seq2	↓0h	6,2094	-0,4799	8,93E-04		↑24h	7,6152	2,2712	1,09E-40
			↓24h	6,2094	-2,3069	4,69E-45	comp33091_c1_seq2	↑0h	6,9661	0,7357	1,82E-05
								↑4h	6,9661	1,7869	1,24E-23
								↑24h	6,9661	2,4001	2,02E-41
Nasvi2EG014864	Unknown	comp28074_c0_seq2	↓24h	3,1998	-1,6454	4,05E-05	comp20374_c0_seq1	↑24h	3,7414	1,1907	1,27E-03
Nasvi2EG014898	LA-related protein	comp32536_c2_seq4	↓0h	2,2381	-1,8165	1,17E-03	comp31788_c1_seq4	↑4h	5,1917	0,8953	8,82E-05
Nasvi2EG015463	Unknown	comp39199_c1_seq16	↓4h	-0,8121	-4,3271	1,58E-03	comp31901_c1_seq1	↑4h	1,8331	2,0393	6,75E-04
								↑24h	1,8331	2,8770	3,95E-06
Nasvi2EG016630	Unknown	comp34555_c0_seq2	↓24h	5,9502	-0,8074	3,76E-08	comp35353_c1_seq1	↑24h	3,9599	1,9505	1,36E-08
Nasvi2EG016764	Pogo transposable element with KRAB domain	comp39896_c1_seq6	↓0h	1,1138	-2,7052	1,03E-04	comp35336_c2_seq12	↑24h	-0,6289	4,6886	6,76E-04
			↓4h	1,1138	-2,6915	4,93E-05					
Nasvi2EG018623	Disulfide-isomerase A3	comp40547_c0_seq1	↓4h	7,6101	-0,3737	1,10E-03	comp36063_c0_seq1	↑24h	8,1445	0,5836	3,01E-04
Nasvi2EG019109	CCR4-NOT transcription complex subunit 7	comp36239_c0_seq3	↓24h	7,2561	-0,4716	9,34E-05	comp31234_c0_seq10	↑24h	2,1320	1,7429	2,26E-03
							comp31234_c0_seq3	↑24h	1,4591	3,1712	1,39E-04
Nasvi2EG019472	Unknown	comp39053_c1_seq11	↓4h	0,5851	-3,6346	1,44E-05	comp31453_c0_seq2	↑4h	-0,7299	6,3406	6,99E-06
		comp39053_c1_seq14	↓4h	0,6211	-2,6901	5,03E-04					
Nasvi2EG020437	Limkain-B1	comp26577_c1_seq1	↓0h	1,9284	-2,1032	1,13E-03	comp18577_c0_seq2	↑24h	5,5353	1,6587	8,47E-14
Nasvi2EG023148	Unknown	comp40553_c0_seq1	↓24h	7,3421	-0,4108	5,66E-04	comp21726_c0_seq1	↑24h	7,8520	0,7327	5,23E-06
Nasvi2EG025129	B-cell lymphoma 3-encoded protein	comp34836_c0_seq1	↓0h	4,2933	-1,1253	8,02E-05	comp27762_c0_seq1	↑0h	4,1749	1,2747	1,04E-04
								↑4h	4,1749	1,2625	1,18E-04

b. Enriched GO-terms among genes with an opposed expression pattern

GO-ID		Term	*P*-value	#Test	#Ref
GO:0006629	P	lipid metabolic process	3,05E-04	6	115
GO:0005811	C	lipid particle	2,72E-03	4	72
GO:0030705	P	cytoskeleton-dependent intracellular transport	1,13E-02	2	19
GO:0006520	P	cellular amino acid metabolic process	1,14E-02	4	110
GO:0016810	F	hydrolase activity, acting on carbon-nitrogen (but not peptide) bonds	3,33E-02	2	35
GO:0051186	P	cofactor metabolic process	4,95E-02	2	44

--Table S8--

Enriched GO terms in antisense transcripts. GO enrichment analyses of antisense transcripts for *N. vitripennis* and *N. giraulti* was done using Blast2go (Fisher's exact test, P < 0.05). The two categories of antisense transcripts were analysed separately: 'antisense2protein' transcripts that have a hit to a *N. vitripennis* protein and 'antisense2sense' transcripts that have a hit to a sense transcript only. Generic GOSlim categories and the Blast2go option 'reduce to most specific terms' were used to limit the number of GO term categories. The enriched GO terms (GO ID and Term), the category of the GO term (C = cellular component, F = molecular function, P = biological process), their *P*-value and the number of genes in the test-set and the reference-set are given.

***N. vitripennis* antisense2protein transcripts**

GO ID	Category	Term	*P*-Value	#Test	#Ref
GO:0005929	C	cilium	6,12E-03	3	13
GO:0006629	P	lipid metabolic process	8,13E-03	8	112
GO:0005578	C	proteinaceous extracellular matrix	2,29E-02	2	8
GO:0007010	P	cytoskeleton organization	2,96E-02	11	229
GO:0004518	F	nuclease activity	3,48E-02	3	27
GO:0005777	C	peroxisome	3,79E-02	2	11
GO:0006259	P	DNA metabolic process	3,98E-02	7	125

***N. vitripennis* antisense2sense transcripts**

GO ID	Category	Term	*P*-Value	#Test	#Ref
GO:0005929	C	cilium	1,85E-02	4	12
GO:0005578	C	proteinaceous extracellular matrix	2,47E-02	3	7
GO:0007610	P	behavior	3,33E-02	24	220
GO:0005886	C	plasma membrane	4,98E-02	34	350

***N. giraulti* antisense2protein transcripts**

GO ID	Category	Term	*P*-Value	#Test	#Ref
GO:0003824	F	catalytic activity	2,31E-02	11	2348
GO:0097159	F	organic cyclic compound binding	2,50E-02	8	1403
GO:1901363	F	heterocyclic compound binding	2,50E-02	8	1403
GO:0010467	P	gene expression	4,03E-02	3	274
GO:0005694	C	chromosome	4,74E-02	2	123

***N. giraulti* antisense2sense transcripts**

GO ID	Category	Term	*P*-Value	#Test	#Ref
GO:0007267	P	cell-cell signaling	4,18E-03	19	216
GO:0009628	P	response to abiotic stimulus	1,35E-02	11	112

GO:0005102	F	receptor binding	1,55E-02	7	56
GO:0006996	P	organelle organization	1,90E-02	32	495
GO:0040007	P	growth	2,17E-02	12	137
GO:0009653	P	anatomical structure morphogenesis	3,46E-02	35	580
GO:0005829	C	cytosol	4,16E-02	11	135
GO:0005794	C	Golgi apparatus	4,64E-02	9	104
GO:0044403	P	symbiosis, encompassing mutualism through parasitism	4,88E-02	3	17

--Table S9--

Antisense transcripts. Differentially expressed antisense transcripts are shown for (a) *N. vitripennis* and (b) *N. giraulti*. The sense transcript and the *N. vitripennis* protein (OGS 2.0) to which the antisense transcripts align are given. It is noted if an antisense transcript aligns to only a protein (p), only a sense transcript (s) or both (b). Also, the differential expression pattern after conditioning is given, compared to naïve expression levels (↑ = upregulated, ↓ = downregulated). The log2-count per million (cpm), log2-fold change (FC), and the level of significance (*P*) are given for the time points at which the transcripts are differentially expressed. In addition, (c) the antisense transcripts that align to known memory genes (see Supplementary table 5) are given.

a. *N. vitripennis* DE antisense transcripts

**Antisense Transcript ID**		**Sense Transcript ID**	**OGS 2.0 ID**	**Gene description**	**Timepoint**	**Log2-cpm**	**Log2-FC**	***P***
comp12119_c0_seq2	p	n.a.	Nasvi2EG000878t1	DNA polymerase η (Pol η)	↑ 0h	-0,65	4,92	6,00E-04
comp39259_c1_seq2	p	n.a.	Nasvi2EG001007t1	α-Esterase 6 (α-Est6)	↑ 0h	0,39	3,33	1,89E-04
					↑ 24h	0,39	2,79	1,19E-03
comp26431_c0_seq1	p	n.a.	Nasvi2EG001246t1	Unknown	↓ 4h	6,21	-0,55	8,28E-05
comp15879_c0_seq1	p	n.a.	Nasvi2EG001294t1	Unknown	↑ 4h	8,03	0,44	3,88E-05
comp41585_c0_seq1	p	n.a.	Nasvi2EG001648t1	Unknown	↓ 24h	6,08	-0,62	1,65E-05
comp40576_c0_seq1	p	n.a.	Nasvi2EG002174t2	Myosin light chain 2 (Mlc2)	↓ 24h	5,09	-1,86	1,42E-19
comp27879_c1_seq1	p	n.a.	Nasvi2EG002278t1	Unknown	↑ 4h	8,10	0,38	3,14E-04
comp41510_c0_seq1	p	n.a.	Nasvi2EG002279t1	Unknown	↑ 0h	5,23	0,85	5,34E-06
					↑ 4h	5,23	0,73	7,95E-05
					↑ 24h	5,23	0,63	7,19E-04
comp35340_c0_seq2	p	n.a.	Nasvi2EG002592t1	Viking (vkg)	↑ 4h	2,36	2,19	4,12E-05
					↑ 24h	2,36	2,42	7,68E-06
comp37392_c1_seq2	p	n.a.	Nasvi2EG002762t1	Unknown	↑ 24h	-0,65	4,36	7,86E-04
comp40161_c0_seq1	p	n.a.	Nasvi2EG003139t2	Coiled-coil domain-containing protein 53 (CCDC53)	↑ 24h	8,15	0,39	2,04E-04

comp37895_c0_seq1	p	n.a.	Nasvi2EG003222t1	Unknown	↓ 4h	-0,96	-4,59	7,92E-04
comp16006_c0_seq1	p	n.a.	Nasvi2EG004018t1	Unknown	↑ 4h	5,94	0,48	1,03E-03
comp41177_c0_seq1	p	n.a.	Nasvi2EG004581t1	Unknown	↑ 4h	7,39	0,42	4,11E-04
comp37243_c1_seq4	p	n.a.	Nasvi2EG005340t1	Estrogen receptor-α binding protein (DNTTIP2)	↓ 24h	-0,52	-4,57	4,14E-04
comp38386_c0_seq2	p	n.a.	Nasvi2EG005833t4	Unknown	↓ 24h	3,81	-2,65	2,65E-15
comp38386_c0_seq3	p	n.a.	Nasvi2EG005838t3	Unknown	↓ 24h	2,44	-3,30	6,35E-09
comp32368_c0_seq1	p	n.a.	Nasvi2EG006084t1	Atrial natriuretic peptide-converting enzyme	↑ 24h	5,19	0,64	6,22E-04
comp45294_c0_seq1	p	n.a.	Nasvi2EG006406t1	Unknown	↑ 4h	6,30	0,43	1,73E-03
comp40040_c1_seq13	p	n.a.	Nasvi2EG008455t1	Unknown	↓ 4h	1,27	-2,17	3,70E-04
comp40040_c1_seq16	p	n.a.	Nasvi2EG008455t1	Unknown	↓ 0h	0,30	-3,38	8,07E-05
					↓ 24h	0,30	-3,50	1,21E-04
comp40040_c1_seq26	p	n.a.	Nasvi2EG008455t1	Unknown	↑ 0h	1,94	4,78	4,45E-10
					↑ 4h	1,94	5,55	1,40E-13
					↑ 24h	1,94	5,13	1,23E-11
comp40040_c1_seq4	p	n.a.	Nasvi2EG008455t1	Unknown	↑ 0h	-0,01	4,71	4,29E-05
					↑ 24h	-0,01	4,18	2,29E-04
comp40040_c1_seq46	p	n.a.	Nasvi2EG008455t1	Unknown	↓ 24h	2,50	-3,09	1,60E-08
comp40040_c1_seq51	p	n.a.	Nasvi2EG008455t1	Unknown	↓ 4h	0,66	-2,99	6,69E-04
comp40040_c1_seq72	p	n.a.	Nasvi2EG008455t1	Unknown	↓ 4h	1,07	-8,98	3,16E-09
					↑ 24h	1,07	2,50	2,29E-04
comp36463_c3_seq1	p	n.a.	Nasvi2EG008589t1	Unknown	↓ 4h	13,80	-0,45	5,58E-64
comp26940_c1_seq1	p	n.a.	Nasvi2EG009169t1	Unknown	↑ 0h	8,53	0,63	4,50E-10
comp39868_c0_seq3	p	n.a.	Nasvi2EG010052t2	Unknown	↑ 24h	0,48	3,50	2,89E-05
comp35162_c1_seq1	p	n.a.	Nasvi2EG010771t1	Unknown	↑ 4h	0,01	2,91	1,58E-03
comp50390_c0_seq1	p	n.a.	Nasvi2EG012180t1	Unknown	↓ 0h	0,59	-2,83	4,25E-04
comp42100_c0_seq1	p	n.a.	Nasvi2EG012476t1	Unknown	↑ 4h	4,79	0,77	7,94E-04
comp42076_c0_seq1	p	n.a.	Nasvi2EG012637t1	Unknown	↑ 0h	5,18	1,30	1,45E-11
					↑ 4h	5,18	1,21	2,75E-10
					↑ 24h	5,18	0,82	2,05E-05
comp39506_c2_seq1	p	n.a.	Nasvi2EG013068t2	Unknown	↓ 0h	4,17	-1,14	1,86E-04

comp14009_c0_seq1	p	n.a.	Nasvi2EG013645t1	Mitochondrial protein	↑ 24h	7,65	0,53	3,22E-06
comp33305_c0_seq1	p	n.a.	Nasvi2EG014392t1	Unknown	↑ 0h	5,08	2,18	1,97E-25
					↑ 4h	5,08	1,69	3,31E-16
					↑ 24h	5,08	1,35	7,10E-11
comp33729_c0_seq1	p	n.a.	Nasvi2EG016290t1	Unknown	↑ 24h	-1,01	10,18	4,06E-06
comp40541_c0_seq1	p	n.a.	Nasvi2EG016509t1	Unknown	↓ 24h	7,78	-0,50	9,93E-06
comp38319_c0_seq5	p	n.a.	Nasvi2EG016602t1	Unknown	↓ 0h	-0,67	-4,77	2,73E-04
					↓ 4h	-0,67	-3,84	1,27E-03
comp24584_c0_seq1	p	n.a.	Nasvi2EG017737t1	Unknown	↓ 0h	-0,07	-7,49	3,54E-04
comp36986_c0_seq1	p	n.a.	Nasvi2EG019969t1	Chromatin assembly factor 1 subunit (Caf1)	↑ 24h	7,47	0,60	2,48E-07
comp39863_c0_seq1	p	n.a.	Nasvi2EG022622t1	Unknown	↓ 4h	-1,15	-5,62	3,70E-04
comp14051_c0_seq1	p	n.a.	Nasvi2EG022711t1	Lactase-phlorizin hydrolase (Lph)	↑ 4h	7,73	0,52	3,99E-06
comp15701_c0_seq1	p	n.a.	Nasvi2EG027514t1	Mitochondrial NADH-ubiquinone oxidoreductase chain 1	↑ 0h	5,26	1,06	4,58E-08
					↑ 24h	5,26	1,38	5,10E-13
comp26945_c0_seq1	p	n.a.	Nasvi2EG034405t1	Unknown	↑ 4h	12,63	0,52	1,03E-32
comp122266_c0_seq1	s	comp39016_c0_seq37	Nasvi2EG000824t7	Intersectin-1 (Itsn1)	↓ 4h	-1,41	-8,36	2,85E-04
comp12119_c0_seq2	b	comp33140_c0_seq3	Nasvi2EG000878t1	DNA polymerase η (Pol η)	↑ 0h	-0,65	4,92	6,00E-04
comp30349_c1_seq2	s	comp38971_c0_seq10	Nasvi2EG001317t1	C-terminal Binding Protein (CtBP)	↑ 4h	1,13	2,12	9,62E-04
comp90866_c0_seq1	s	comp32601_c0_seq1	Nasvi2EG001683t1	Unknown	↓ 4h	-0,85	-7,52	9,83E-04
comp40576_c0_seq1	b	comp14182_c0_seq1	Nasvi2EG002174t2	Myosin light chain 2 (Mlc2)	↓ 24h	5,09	-1,86	1,42E-19
comp10600_c0_seq1	s	comp35366_c0_seq6	Nasvi2EG002377t1	Rab-26	↑ 24h	-1,24	5,11	9,85E-04
comp35340_c0_seq2	b	comp41119_c0_seq1	Nasvi2EG002592t1	Viking (vkg)	↑ 4h	2,36	2,19	4,12E-05
					↑ 24h	2,36	2,42	7,68E-06
comp144171_c0_seq1	s	comp38870_c0_seq19	Nasvi2EG002897t1	Alan shepard	↑ 0h	-0,77	9,52	1,07E-05
					↑ 4h	-0,77	8,50	1,23E-04
comp148455_c0_seq1	s	comp26213_c0_seq3	Nasvi2EG004025t3	Ubiquitin conjugation factor E4 A (UBE4A)	↑ 0h	0,21	4,51	1,19E-05
					↑ 4h	0,21	5,08	6,89E-07
comp245225_c0_seq1	s	comp26213_c0_seq3	Nasvi2EG004025t3	Ubiquitin conjugation factor E4 A (UBE4A)	↑ 0h	0,19	3,52	2,05E-04
					↑ 4h	0,19	4,16	9,74E-06
comp40127_c2_seq1	s	comp43375_c0_seq1	Nasvi2EG004081t1	Sir2-like protein 4 (Sirt4)	↑ 24h	9,81	0,36	1,42E-05

comp27593_c1_seq1	s	comp44977_c0_seq1	Nasvi2EG005962t3	DDB1- and CUL4-associated factor 7 (DCAF7)	↑ 0h	3,92	1,31	2,15E-05
comp45404_c0_seq1	s	comp14062_c0_seq1	Nasvi2EG005970t2	Arginine kinase (Argk)	↓ 24h	1,82	-1,81	2,00E-03
comp170983_c0_seq1	s	comp26764_c0_seq1	Nasvi2EG007077t1	Unknown	↑ 0h	0,37	5,07	4,74E-07
comp50970_c0_seq1	s	comp33484_c1_seq2	Nasvi2EG007097t1	B-type amino acid transporter 1	↓ 0h	0,47	-2,91	5,21E-04
comp4527173_c0_seq1	s	comp44329_c0_seq1	Nasvi2EG007254t1	Nuclear pore complex protein 153 (Nup153)	↑ 0h	-0,22	7,75	9,58E-04
					↑ 4h	-0,22	8,22	3,43E-04
					↑ 24h	-0,22	11,07	7,11E-07
comp38256_c4_seq1	s	comp39615_c8_seq6	Nasvi2EG008154t1	Golgi phosphoprotein 3	↑ 0h	6,35	1,36	1,09E-22
					↑ 4h	6,35	0,57	3,63E-05
comp171469_c0_seq1	s	comp38255_c2_seq6	Nasvi2EG008479t1	Hippocampus abundant transcript 1 (Hiat1)	↑ 0h	-0,83	8,25	8,77E-04
					↑ 4h	-0,83	7,46	1,43E-03
					↑ 24h	-0,83	8,15	3,84E-04
comp483118_c0_seq1	s	comp38080_c0_seq26	Nasvi2EG008626t2	Phosphatidylinositol 3-kinase regulatory subunit alpha	↑ 0h	-0,35	8,20	9,96E-05
					↑ 4h	-0,35	7,62	4,96E-04
					↑ 24h	-0,35	10,09	3,26E-07
comp1768165_c0_seq1	s	comp38641_c1_seq4	Nasvi2EG009527t2	EH domain-binding protein 1 (Ehbp1)	↑ 4h	-1,18	7,80	8,31E-04
comp14746_c0_seq1	s	comp46035_c0_seq1	Nasvi2EG009912t1	Glycosyltransferase family 4 protein (DPAGT1)	↓ 0h	0,06	-9,32	2,44E-07
comp50390_c0_seq1	b	comp42985_c0_seq1	Nasvi2EG012180t1	Unknown	↓ 0h	0,59	-2,83	4,25E-04
comp29402_c0_seq1	s	comp39228_c0_seq32	Nasvi2EG013237t2	Still life (sif)	↑ 0h	-0,77	7,71	2,30E-04
					↑ 4h	-0,77	8,24	5,11E-05
comp34440_c0_seq1	s	comp35284_c0_seq1	Nasvi2EG013374t2	Sarcalumenin	↓ 24h	-1,20	-7,90	3,86E-04
comp14009_c0_seq1	b	comp32300_c0_seq4	Nasvi2EG013645t1	Mitochondrial protein	↑ 24h	7,65	0,53	3,22E-06
comp60714_c0_seq1	s	comp14354_c0_seq1	Nasvi2EG013795t1	N-acetylglucosamine-1-phosphotransferase subunits α/β	↓ 24h	-0,71	-7,76	7,10E-04
comp41709_c0_seq1	s	comp42747_c0_seq1	Nasvi2EG015630t1	Coproporphyrinogen-III oxidase	↑ 4h	5,57	0,86	1,04E-07
					↑ 24h	5,57	0,66	4,69E-05
comp33729_c0_seq1	b	comp40144_c0_seq1	Nasvi2EG016290t1	Unknown	↑ 24h	-1,01	10,18	4,06E-06
comp26920_c1_seq1	s	comp14423_c0_seq2	Nasvi2EG019953t1	SH2 ankyrin repeat kinase (shark)	↑ 0h	6,91	0,85	1,93E-11
comp103601_c0_seq1	s	comp39514_c1_seq6	Nasvi2EG020485t2	Fasciclin-1	↑ 4h	-1,00	10,34	4,26E-05
comp40728_c0_seq1	s	comp38591_c4_seq3	Nasvi2EG025482t1	Proton-coupled amino acid transporter 4 (Pat4)	↓ 4h	5,41	-0,56	8,47E-04

b. *N. giraulti* DE antisense transcripts

Antisense Transcript ID		Sense Transcript ID	OGS 2.0 ID	Gene description	Timepoint	Log2-cpm	Log2-FC	*P*
comp38357_c0_seq1	p	n.a.	Nasvi2EG003943t4	Myosin II heavy chain	↓ 24h	3,47	-1,52	7,20E-05
comp22039_c0_seq1	p	n.a.	Nasvi2EG007369t1	Inosine-uridine preferring nucleoside hydrolase	↓ 0h	4,51	-1,03	4,52E-04
comp29795_c0_seq2	p	n.a.	Nasvi2EG016290t1	Unknown	↑ 4h	-0,36	4,43	3,21E-04
					↑ 24h	-0,36	4,36	3,45E-04
comp347886_c0_seq1	s	comp29532_c1_seq2	Nasvi2EG000786t1	Cold-shock DNA-binding protein	↑ 0h	-0,09	4,92	2,24E-05
					↑ 4h	-0,09	3,83	1,32E-03
					↑ 24h	-0,09	3,79	5,35E-04
comp569819_c0_seq1	s	comp34287_c1_seq37	Nasvi2EG001295t1	Voltage-dependent L-type calcium channel subunit β2	↑ 0h	-1,06	9,23	1,20E-05
					↑ 4h	-1,06	8,08	2,07E-04
comp225902_c0_seq1	s	comp16263_c0_seq1	Nasvi2EG002804t1	Unknown	↑ 0h	0,40	8,01	5,04E-06
					↑ 4h	0,40	10,28	3,01E-11
					↑ 24h	0,40	8,97	5,37E-08
comp67785_c0_seq1	s	comp37138_c0_seq1	Nasvi2EG002805t1	Unknown	↑ 0h	0,55	4,17	7,24E-07
					↑ 4h	0,55	3,88	4,95E-06
comp27193_c0_seq1	s	comp22661_c0_seq2	Nasvi2EG002825t2	Peroxisomal acyl-coenzyme A oxidase 3 (Acox3)	↑ 24h	2,84	2,20	5,79E-06
comp169896_c0_seq1	s	comp22661_c0_seq2	Nasvi2EG002825t2	Peroxisomal acyl-coenzyme A oxidase 3 (Acox3)	↑ 24h	1,42	2,36	8,29E-05
comp187620_c0_seq1	s	comp22661_c0_seq2	Nasvi2EG002825t2	Peroxisomal acyl-coenzyme A oxidase 3 (Acox3)	↑ 24h	1,45	2,16	2,91E-04
comp38357_c0_seq1	b	comp35611_c0_seq1	Nasvi2EG003943t4	Myosin II heavy chain	↓ 24h	3,47	-1,52	7,20E-05
comp27325_c0_seq1	s	comp31105_c0_seq2	Nasvi2EG004970t2	Mi-2	↓ 4h	-1,02	-8,92	1,75E-05
comp35917_c0_seq1	s	comp32548_c0_seq4	Nasvi2EG005631t1	Vacuolar protein sorting-associated protein 62 (VPS62)	↓ 4h	7,71	-0,78	1,03E-06
					↓ 24h	7,71	-0,65	4,58E-05
comp28725_c0_seq1	s	comp35314_c2_seq50	Nasvi2EG007625t1	RalBP1-associated Eps domain-containing protein 2 (Reps2)	↓ 24h	5,60	-0,66	1,09E-03
comp424968_c0_seq1	s	comp21783_c2_seq1	Nasvi2EG008589t1	Unknown	↑ 0h	1,31	2,75	2,89E-05
					↑ 4h	1,31	3,03	8,30E-06
					↑ 24h	1,31	2,32	4,28E-04
comp3195_c0_seq1	s	comp37565_c0_seq1	Nasvi2EG009042t1	Unknown	↑ 24h	2,35	1,90	3,78E-04
comp509400_c0_seq1	s	comp37565_c0_seq1	Nasvi2EG009042t1	Unknown	↑ 24h	2,12	1,94	4,82E-04

comp29704_c0_seq1	s	comp35919_c0_seq1	Nasvi2EG009949t1	Unknown	↑ 4h	1,20	2,40	1,39E-04
comp29619_c0_seq1	s	comp16892_c0_seq1	Nasvi2EG011945t1	Unknown	↑ 0h	-1,25	8,21	2,80E-04
					↑ 4h	-1,25	8,38	2,02E-04
comp22419_c0_seq1	s	comp30627_c0_seq2	Nasvi2EG012162t1	AN1-type zinc finger protein 5	↑ 0h	0,36	2,86	6,77E-04
comp32497_c1_seq1	s	comp32497_c0_seq3	Nasvi2EG015142t1	Neuronal synaptobrevin (n-syb)	↑ 0h	-0,20	8,68	1,46E-06
					↑ 4h	-0,20	9,51	7,14E-08
					↑ 24h	-0,20	8,06	1,79E-05
comp29795_c0_seq2	b	comp35558_c0_seq1	Nasvi2EG016290t1	Unknown	↑ 4h	-0,36	4,43	3,21E-04
					↑ 24h	-0,36	4,36	3,45E-04
comp60918_c0_seq1	s	comp22214_c0_seq2	Nasvi2EG017292t1	Alkaline sphingomyelin phosphodiesterase (aSMase)	↑ 0h	0,36	2,97	3,83E-04
comp27107_c0_seq1	s	comp29331_c3_seq2	Nasvi2EG017578t2	Bromo adjacent homology domain-containing 1 protein (BAHD1)	↑ 0h	0,24	3,36	2,58E-04
					↑ 4h	0,24	3,35	3,49E-04
comp36012_c0_seq1	s	comp33932_c0_seq23	Nasvi2EG020114t1	Metallophosphoesterase domain-containing protein 1 (Mpped1)	↓ 4h	6,88	-0,70	2,61E-05
comp21577_c0_seq1	s	comp35498_c0_seq22	Nasvi2EG020143t1	Staphylococcal nuclease domain-containing protein 1 (Snd1)	↑ 4h	0,81	2,72	1,97E-04
comp289803_c0_seq1	s	comp16755_c0_seq2	Nasvi2EG020600t1	Cytochrome P450 CYP4AB2	↑ 0h	0,07	3,15	5,69E-04
comp34789_c3_seq1	s	comp34789_c1_seq2	Nasvi2EG023320t1	Solute carrier family 35 member F3 (Slc35f3)	↓ 24h	-0,71	-8,60	3,53E-05
comp26624_c0_seq1	s	comp21764_c0_seq2	Nasvi2EG024737t1	Rad50	↑ 4h	0,45	2,60	1,48E-03

c. antisense transcripts that align to known memory genes

Gene/transgene	Gene description	Antisense Transcript ID *N. vitripennis*	*N. giraulti*
CAMKI	calcium/calmodulin dependent kinase I	comp35472_c2_seq1 (s)	
CAMKII	calcium/calmodulin dependent kinase II	comp45169_c0_seq1 (s)	
Clumsy	glutamate receptor	comp40019_c1_seq8 (b)	
		comp40019_c1_seq11 (b)	
Crammer	trans-inhibitor of cathepsins	comp36751_c0_seq2 (b)	
Dunce	cAMP phosphodiesterase	comp24558_c0_seq1 (s)	comp281362_c0_seq1 (s)
Fasciclin 1	neural cell adhesion molecule	comp103601_c0_seq1 (s)	
GluCl	glutamate-gated chloride channel	comp37299_c0_seq1 (s)	
Leonardo	14-3-3 zeta protein family		comp18485_c0_seq1 (b)
NF1	ras GTPase activating protein	comp43282_c0_seq1 (s)	
Octβ2R	octopamine β2 receptor	comp39256_c0_seq1 (s)	
Radish	Rap GTPase activating protein		comp28992_c3_seq1 (s)
S6KII	ribosomal S6 kinase (RSK)	comp14791_c0_seq1 (s)	comp130445_c0_seq1 (s)
Tdc1	tyrosine decarboxylase 1	comp95025_c0_seq1 (s)	
Tequila	neurotrypsin	comp38985_c0_seq3 (s)	
		comp38985_c0_seq4 (s)	
